# Formation of Blood Neutrophil Extracellular Traps Increases the Mastitis Risk of Dairy Cows During the Transition Period

**DOI:** 10.3389/fimmu.2022.880578

**Published:** 2022-04-27

**Authors:** Lu-Yi Jiang, Hui-Zeng Sun, Ruo-Wei Guan, Fushan Shi, Feng-Qi Zhao, Jian-Xin Liu

**Affiliations:** ^1^Institute of Dairy Science, College of Animal Sciences, Zhejiang University, Hangzhou, China; ^2^Department of Veterinary Science, College of Animal Sciences, Zhejiang University, Hangzhou, China; ^3^Department of Animal & Veterinary Sciences, University of Vermont, Burlington, MA, United States

**Keywords:** neutrophil extracellular traps, cell cycle, somatic cell count, mastitis risk, transition dairy cow

## Abstract

The current study was conducted to analyze the functions of blood neutrophils in transition cows and their association with postpartum mastitis risk as indicated by somatic cell counts (SCCs) in milk. Seventy-six healthy Holstein dairy cows were monitored from Week 4 prepartum to Week 4 postpartum. Five dairy cows with low SCCs (38 ± 6.0 × 10^3^/mL) and five with high SCCs (3,753 ± 570.0 × 10^3^/mL) were selected based on milk SCCs during the first three weeks of lactation. At Week 1 pre- and postpartum, serum samples were obtained from each cow to measure neutrophil extracellular trap (NET)-related variables, and blood neutrophils were collected for transcriptome analysis by RNA sequencing. The serum concentration of NETs was significantly higher (*P* < 0.05) in cows with high SCCs than in cows with low SCCs (36.5 ± 2.92 vs. 18.4 ± 1.73 ng/mL). The transcriptomic analysis revealed that the transcriptome differences in neutrophils between high- and low-SCC cows were mainly in cell cycle-related pathways (42.6%), including the cell cycle, DNA damage, and chromosomal conformation, at Week 1 prepartum. The hub genes of these pathways were mainly involved in both the cell cycle and NETosis. These results indicated that the formation of NETs in the blood of transition dairy cows was different between cows with low and high SCCs, which may be used as a potential indicator for the prognosis of postpartum mastitis risk and management strategies of perinatal dairy cows.

## Introduction

Dairy cows are accompanied by marked physiologic inflammatory adjustments mastitis during the transition period (from 3 weeks prepartum to 3 weeks postpartum). It has become firmly established that the inflammatory response and immune activation of the mammary gland are a normal component of transition cow biology (1, 2). However, if inflammation becomes pathological, it increases the mastitis risk, leading to reductions in lactation performance and milk quality (3). Mastitis-related immune responses are complex biological processes involving immune cells, mammary epithelial cells, and endothelial cells in blood vessels (4). The somatic cell count (SCC) is the total number of somatic cells in milk, includes shed mammary epithelial cells and white blood cells and is usually used as a biomarker of mastitis risk (1). SCC is closely linked to the immune status in the mammary gland in lactating cows (5). Therefore, investigating the correlation between the SCC and immune status during the transition period will provide novel insights and potential strategies to improve immune function and milk quality in dairy cows.

In the immune responses in dairy cows (6), neutrophils are most of the leukocytes that migrate into the mammary gland to defend against infection, and these cells are also a major component of the SCC (7). The massive recruitment of blood neutrophils to infection sites is a hallmark of infectious diseases, such as mastitis (6). In our previous work, the numbers of neutrophils tended to be higher in dairy cows with high SCCs than in those with low SCCs during the transition period (8). In addition, functional variations in neutrophils have been observed in a variety of diseases, including inflammatory diseases in humans (9) and mastitis in dairy cows (10). However, the relationship between neutrophil functions and SCCs in dairy cows during the transition period has not been studied. The migration of neutrophils into the mammary gland mainly depends on adhesion, migration, chemotaxis, phagocytosis, and antimicrobial actions (11). Neutrophil extracellular traps (NETs) are large, extracellular, web-like structures composed of cytosolic and granule proteins that are assembled on a scaffold of decondensed chromatin (12). NETosis is a novel microbe-eliminating process of neutrophils and has recently received much attention (13). The formation of NETs is mediated by reactive oxygen species (ROS) and is associated with mitogenic reactivation of cell cycle regulators (14, 15). Previous studies have shown that serum ROS concentrations are decreased in low-SCC dairy cows but are increased in high-SCC cows from Week 1 prepartum to Week 1 postpartum (8), suggesting that high ROS concentrations in dairy cows with high mastitis risk may mediate the formation of NETs. Changes in neutrophil phagocytosis, antimicrobial actions, chemotaxis, and adhesion are associated with mastitis in dairy cows (8, 16). However, which neutrophil functions are mostly related to mastitis in dairy cows needs to be studied.

Transcriptomics is a powerful method for exploring the temporal and spatial specificity of gene expression (17). RNA sequencing (RNA-Seq)-based transcriptomics can detect whole-genome expression changes in different conditions and can provide a holistic understanding of the relationship between blood neutrophil functions and mastitis risk in cows during the transition period (18). We hypothesized that the formation of NETs in the blood of transition dairy cows is different between cows with low and high SCCs, which may be used as an indicator of mastitis risk in dairy cows during the transition period. This work aimed to study the relationship between neutrophil functions and SCC changes in postpartum milk.

## Materials and Methods

All animal experimental procedures were approved by the Animal Care Committee of Zhejiang University (Hangzhou, Zhejiang, China) and were in accordance with the university’s guidelines for animal research (No. ZJU20200031).

### Experimental Design and Sampling

The animals used in this study were selected from 76 Holstein cows used in a previous study (8). In order to investigate the mechanism behind the SCC variations, five cows with low SCCs (38 ± 6.0) × 10^3^/mL and five with high SCCs (3,753 ± 570.0) × 10^3^/mL were selected for this study based on the average SCCs during the first three weeks postpartum. Briefly, all ten cows had similar parity (2.60 ± 0.221), and they were in health monitored weekly and had no clinical symptoms records during this study. They were housed in individual tie stalls, bedded with sawdust, and had free access to water. Diet that was designed to meet the nutrient requirements of dry and early lactating cows (19) was offered as total mixed rations to allow approximately 5% orts three times daily (at 06:30, 13:00, 18:30). After the experiment, the cows with high SCC were transferred to the unified area of the dairy farm for antibiotics treatment. There were four metabolic clusters in this study: prepartum (Week 1 prepartum), low SCC (PR-low); postpartum (Week 1 postpartum), low SCC (PP-low); prepartum, high SCC (PR-high); and postpartum, high SCC (PP-high). Blood and milk samples were collected from the cows as described previously (8). Briefly, blood samples were collected from the tail vein at Week 1 pre- and postpartum. One sample was stored at 4°C until the serum separated, the other samples were used to isolate neutrophils by gradient centrifugation, and the isolated neutrophils were identified by flow cytometry (20). The purity of neutrophils was approximately 97%. The serum and neutrophil samples were stored at -80°C for further analyses. Milk samples were collected three weeks postpartum, and milk composition was analyzed (8).

### Serum Cytokines and Oxidative Stress Variables

The serum concentrations of interleukin-1β (IL-1β, #SEKB-0363), interleukin-8 (IL-8, #SEKB-0366), and interleukin-17 (IL-17, #SEKB-0006) were measured using corresponding enzyme-linked immunosorbent assay (ELISA) kits (Beijing Solarbio Science & Technology, Beijing, China) (21).

The serum ROS concentration was measured with a fluorescent dichlorofluorescein-diacetate (DCFH-DA) probe provided by an ROS kit (#E004-1-1, Nanjing Jiancheng Bioengineering Institute, Nanjing, China) (22). Lipid peroxidation in serum, which is expressed as the malondialdehyde (MDA) concentration, was measured using an MDA assay kit (#A003-1-2, Nanjing Jiancheng Bioengineering Institute) (23). Protein oxidation level, which is expressed as the concentration of protein carbonyls (PC), was measured according to a described previously method with a PC kit (#BC1275, Beijing Solarbio Science & Technology) (24). The level of 8-hydroxy-2-deoxyguanosine (8-OHdG) was measured with an ELISA kit (#E-EL-0028, Elabscience Biotechnology, Wuhan, China) (25).

### Serum Neutrophil Extracellular Trap-Related Indices

The blood samples were clotted in serum tubes for 2 h at room temperature before being centrifuged at 1,000 × g for 20 min. Freshly prepared serum was assayed immediately or stored in aliquots at -80 °C for further analysis. The NET level of serum samples was measured according to a previously described method (26). The serum myeloperoxidase (MPO)-DNA complexes were determined as a marker of NETs (27, 28). Briefly, the antibodies (anti-MPO, 1:2,000 dilution in sterile PBS) were captured in 96-well high-binding capacity ELISA microplates and incubated overnight at 4°C. In addition, the plates were washed with PBS-Tween 20, blocked with 5% BSA (200 μL/well), incubated for another 2 h at room temperature, and washed with PBS-Tween 20 again. After that, the serum samples were incubated in ELISA plates overnight at 4°C. The plates were washed with PBS-Tween 20, and the detection antibody (HRP-labeled anti-dsDNA, 1:500, mouse, 50 μL/well) was added and incubated for 1 h in the dark at room temperature. The plates were then washed again with PBS-Tween 20, TMB peroxidase substrate was added to each well and incubated for 30 min (50 μL/well), and the reaction was terminated by the addition of 1 M HCl (50 μL/well). The NETs inter-assay CV is 10% and intra-assay CV is 15%. The absorbance was measured using a microplate photometer (Thermo Fisher Scientific, Tokyo, Japan) at 450 nm.

The concentration of serum myeloperoxidase (MPO, #A044-1-1, inter-assay CV is 1.3% and intra-assay CV is 1.3%) was measured using an MPO kit (Nanjing Jiancheng Bioengineering Institute) and the contents of deoxyribonuclease I (DNase I, #CK-EN77261, inter-assay CV is 10% and intra-assay CV is 15%) and neutrophil elastase (NE, #CK-EN78022, inter-assay CV is 10% and intra-assay CV is 15%) in serum samples were determined using corresponding ELISA kits (Quanzhou Ruixin Science & Technology, Quanzhou, China) (29, 30).

### RNA Isolation, Library Construction, RNA Sequencing and Bioinformatics Analysis

Total RNA was isolated from blood neutrophils using TRIzol reagent (TaKaRa, Dalian, China) according to the manufacturer’s instructions (31), and RNA quality was validated on an Agilent Technologies 2200 bioanalyzer. The library was amplified with phi29 (Thermo Fisher Scientific, Waltham, MA, USA) and sequenced using the Illumina HiSeq X ten platform (32). Clean reads were obtained by removing low-quality sequences (more than 30% of < Q20 bases), reads with more than 10% unknown nucleotides (N), and adapters. The clean reads were aligned with the bovine genome (ARS-UCD1.2), which was downloaded from the ENSEMBL database (http://www.ensembl.org/index.html), to assemble transcripts using salmon (v 1.4.0) (33). The gene expression levels were normalized using the fragments per kilobase of transcript per million fragments (FPKM) method.

### Gene Coexpression Network Analysis and Expression Level

The hybrid coexpression networks of blood neutrophils were constructed using the weighted gene coexpression network analysis (WGCNA) package in R with FPKM data (34). To obtain coexpression patterns, we set the minimum module size to 30 genes, the deep split to 2, and the minimum height for merging modules to 0.25. Each module was summarized by an eigengene, which was the first principal component of the scaled module expression. To obtain cleaner modules, we defined the module membership measure (also known as module eigengene-based connectivity kME) as the correlation between gene expression values and the module eigengene. Genes with kME > 0.7 were cut off to obtain the hub genes. Moreover, the hub genes of the brown module were submitted to analyze the cell cycle pathway with Cytoscape software (v3.7.2).

Quantitative real-time PCR (qPCR) was performed as described by Mohamed et al. (31). Total RNA was isolated from blood neutrophils using TRIzol reagent (TaKaRa) and then reverse-transcribed using a commercial kit (Perfect Real Time, SYBR^@^ PrimeScript ™, TaKaRa) according to the manufacturer’s instructions. The mRNA expression of genes involved in the cell cycle pathway, including *TP53*, *CDK1*, *CDK2*, *CDK4*, *CCNA2*, *CCNB3*, *CCND1*, *CCNE1*, *CDC25A*, *DP1*, *C-Myc*, *ORC1*, *ORC2*, *ORC4*, *MCM3*, *MCM5*, and *MCM6*, was analyzed (35). The fold-changes in the expression of individual genes were calculated according to the 2^-ΔΔCt^ method (35), and *β-actin* gene expression was used as an internal standard. The primer sequences used in qPCR are shown in **Table S1**.

### Functional Analysis of Differentially Expressed Genes in Prepartum Cows

Deseq2 software (V1.4.5) was used to identify the differentially expressed genes (DEGs) in blood neutrophils between PR-low and PR-high cows (FDR < 0.05 and |log2FoldChange| ≥ 1) (36).

The data were annotated using the gene ontology (GO) database (http://www.geneontology.org/) by the hypergeometric test to examine the biological mechanisms and pathways of these genes. The GO terms were considered significant when the *P* value was less than 0.05. Pathway analyses of these data were also performed using the Kyoto Encyclopedia of Genes and Genomes (KEGG) database (http://www.genome.jp/kegg/), and those with *P* values less than 0.05 were considered to be significantly different. The data were used in gene set enrichment analysis (GSEA), which is a computational pathway analysis tool that determines if a given set of manually curated genes shows statistically significant, concordant differences between two biological states (37) and is accessible at http://www.broadinstitute.org/-gsea/index.jsp. The *P* values and false discovery rate (FDR) for the enrichment scores of the gene set were calculated based on 1,000 gene set permutations (|NES| > 1, *P* < 0.05, FDR < 0.05).

### Statistical Analysis

This study was performed a 2 × 2 experimental design with health status (Hs, low SCC vs. high SCC) and sampling week (Wk, -1 Wk vs. +1 Wk) as independent factors, interaction (Hs × Wk) as fixed effects, and the dairy cow as the dependent variable. The data (except RNA-Seq data) of different indices were analyzed with the repeated measurements mixed procedure (PROC MIXED) by Statistical Analysis System software (version 9.1; SAS Institute, Inc., Cary, NC, USA). The data are expressed as mean ± standard error of the mean (SEM).

## Results

### Formation of Blood Neutrophil Extracellular Traps in Transition Dairy Cows

The serum cytokine and oxidative stress variable results are shown in **Table 1**. Compared with those in cows with low SCCs, the levels of serum cytokines (IL-1β, IL-8, and IL-17) and oxidative stress variables (ROS, MDA, 8-OHDG, and PC) were significantly higher (*P* < 0.05) in cows with high SCCs. The serum levels of IL-1β, IL-17, MDA, 8-OHDG, and PC were significantly higher (*P* < 0.05) postpartum than prepartum. No significant difference was found in IL-8 or ROS levels between prepartum and postpartum cows. The interaction effects between SCC levels and time (Hs × Wk) were significant for MDA and PC (*P* < 0.05).

**Table 1:** Serum concentrations of cytokines and oxidative stress variables in dairy cows with low and high milk somatic cell counts (SCCs)^1^ at Week 1 pre- (-1 Wk) and postpartum (+1 Wk).

Items^3^	-1 Wk		+1 Wk			*P*-value^2^		
	Low SCC	High SCC	Low SCC	High SCC	SEM	Hs	Wk	Hs × Wk
*Cytokines*								
IL-1β (pg/mL)	149^c^	294^ab^	236^b^	357^a^	22.5	<0.01	<0.01	0.58
IL-8 (pg/mL)	81^b^	126^ab^	94^b^	162^a^	8.6	<0.01	0.09	0.40
IL-17 (pg/mL)	196^c^	315^b^	220^c^	383^a^	19.4	<0.01	0.03	0.27
*Oxidative stress variables*								
ROS (IU/mL)	274^c^	329^ab^	251^c^	337^a^	11.3	<0.01	0.72	0.43
MDA (nmol/mL)	0.66^c^	1.68^b^	0.70^c^	3.05^a^	0.229	<0.01	<0.01	<0.01
8-OHDG (ng/mL)	97^b^	111^b^	115^b^	165^a^	7.0	<0.01	<0.01	0.10
PC (umol/mL ×10^-3^)	43.5^bc^	51.3^b^	40.9^c^	73.5^a^	3.17	<0.01	<0.01	<0.01

^1^The data are expressed as the mean ± SEM, n = 5 cows per group. Different superscripts (a, b, and c) represent significant differences (P < 0.05).

^2^Hs, health status; Wk, sampling week; Hs × Wk, interaction between health status and sampling week.

^3^IL-1β , interleukin-1β; IL-8, interleukin-8; IL-17, interleukin-17; ROS, reactive oxygen species; 8-OHDG, 8-hydroxy-2 deoxyguanosine; PC, protein carbonyl.

The serum concentrations of NET-related variables are shown in **Table 2**. The levels of NETs (36.5 ± 2.92 vs. 18.4 ± 1.73 ng/mL) and the main functional components (MPO and NE) of NETs were significantly higher (*P* < 0.05) in the high-SCC cows than in the low-SCC cows, whereas NET marker (DNase I) concentration was significantly lower (*P* < 0.05). The levels of NETs (32.2 ± 4.01 vs. 22.7 ± 2.97 ng/mL) and their main functional components (MPO and NE) were significantly higher (*P* < 0.05) postpartum than prepartum. No significant difference was found in DNase I level between prepartum and postpartum cows. The interaction effects (Hs × Wk) were found to be significant for the concentration of serum MPO (*P* < 0.05).

**Table 2:** Serum concentrations of NET-related indices in dairy cows with low and high somatic cell counts (SCCs)^1^ at Week 1 pre- (-1 Wk) and postpartum (+1 Wk).

Items^3^	-1 Wk		+1 Wk			*P*-value ^2^		
	Low SCC	High SCC	Low SCC	High SCC	SEM	Hs	Wk	Hs × Wk
NETs (ng/mL)	15.4^c^	30.1^b^	21.4^c^	43.0^a^	2.66	<0.01	<0.01	0.16
DNase I (U/L)	69.0^b^	38.0^a^	67.0^b^	37.7^a^	3.92	<0.01	0.76	0.81
MPO (U/L)	97.6^c^	157^b^	114^bc^	229^a^	13.4	<0.01	<0.01	0.04
NE (U/L)	101^c^	170^b^	152^b^	201^a^	8.9	<0.01	<0.01	0.11

^1^The data are expressed as the mean ± SEM, n = 5 cows per group. Different superscripts (a, b, and c) represent significant differences (P < 0.05).

^2^Hs, health status; Wk, sampling week; Hs × Wk, interaction between health status and sampling week.

^3^NETs, neutrophil extracellular traps; MPO, myeloperoxidase; NE, neutrophil elastase.

### RNA Sequencing Analysis of Blood Neutrophils

More than 42.8 million raw reads were generated by RNA-Seq from a total of 20 blood neutrophil samples: five samples each from the PR-low (44.9 ± 1.58), PP-low (45.9 ± 0.83), PR-high (45.7 ± 1.45), and PP-high (48.0 ± 2.30) groups (**Table S2**). More than 38.6 million clean reads were obtained from these raw reads after low-quality and adaptor sequences were filtered out, with a sequencing error rate of less than 1%. More than 88.1% of reads were mapped to the bovine genome (ARS-UCD1.2) for the PR-low (90.0 ± 0.40%), PP-low (90.2 ± 0.42%), PR-high (89.6 ± 1.01%), and PP-high (89.2 ± 1.10%) groups (**Table S2**). Among these clean reads, more than 89.7% had quality scores with a ratio of Q30 (a base quality >30 and error rate <0.001). No GC bias was found.

Principal component analysis (PCA) revealed a relatively clear distinction between the transcriptomes of blood neutrophils from the low-SCC and high-SCC cows, with the first two principal components showing 59.8% variance (**Figure S1A**). The boxplots of FPKM values of blood neutrophil transcripts revealed that sequencing depth and gene length were at the same levels (**Figure S1B**).

### Neutrophil Functions Related to Somatic Cell Count Variations

**Figure 1:**
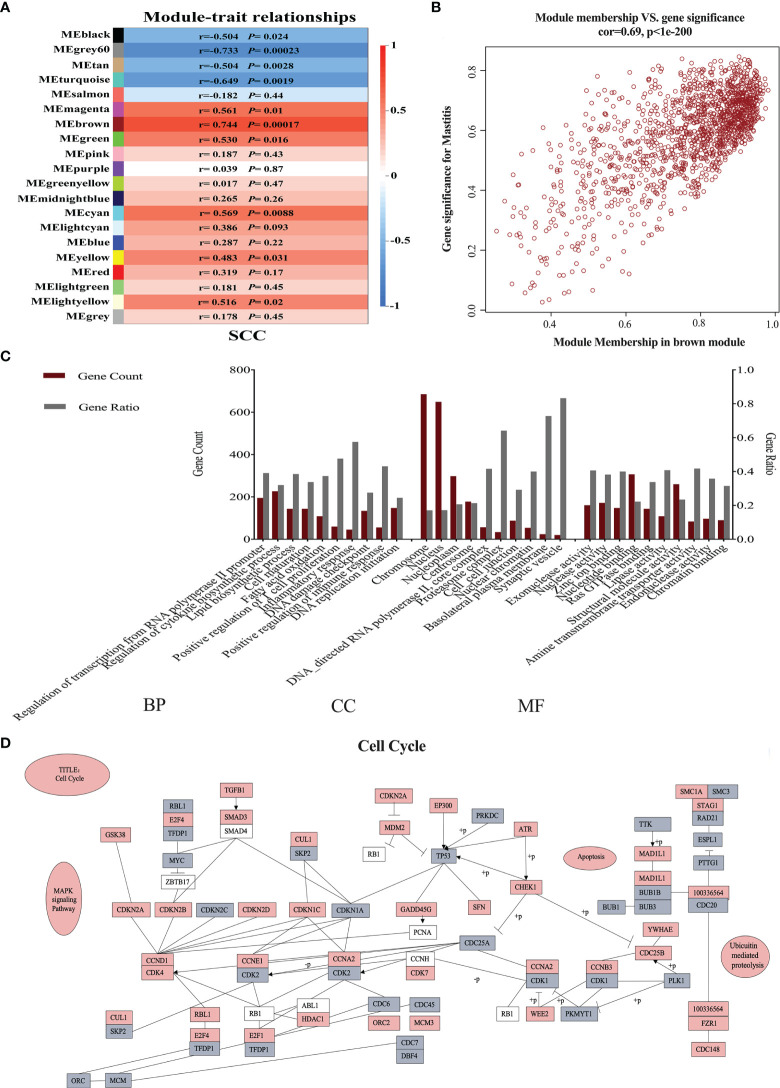
Neutrophil weighted gene coexpression network analysis in dairy cows with low and high somatic cell counts (SCCs) at Week 1 pre- and postpartum. **(A)** The relationship between coexpressed modules and SCCs. **(B)** Gene distribution map in the brown module is shown in brown in **(A)**. **(C)** Gene ontology analysis of hub genes in the brown module. **(D)** The hub genes involved in the cell cycle. Gray indicates downregulated genes, and red represents upregulated genes. BP, biological process; CC, cellular component; MF, molecular function.

A total of 20 gene modules related to SCC levels were identified (**Figure 1A**). Among these modules, the MEbrown module exhibited the highest correlation (r-value = 0.744) and the lowest *P* value (0.00017). A total of 1,439 genes were involved in the MEbrown module (r = 0.69, *P* < 0.05, **Figure 1B**). These genes were analyzed for GO enrichment. The top 10 biological process (BP), cellular component (CC), and molecular function (MF) GO terms with the smallest *P* values are shown in **Figure 1C**. The enriched GO terms in BP were mainly related to immunity (40%), cell cycle (20%), energy metabolism (20%), cell maturation (10%), and transcription (10%). The GO terms in CC included cell cycle (80%), cell connection (10%), and cell signal transduction (10%). The GO terms in MF included cell cycle (70%), signal transduction (10%), energy metabolism (10%), and structural molecule activity (10%). The hub genes of the MEbrown module were subjected to cell cycle pathway analysis. The detailed regulation of these genes in cell cycle pathways is shown in **Figure 1D**.

**Figure 2:**
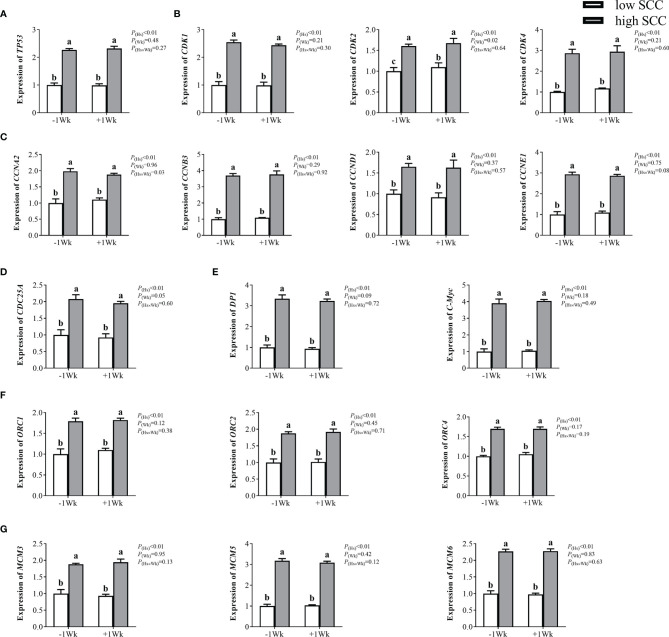
The mRNA expression of genes involved in the cell cycle in neutrophils from dairy cows with low and high somatic cell counts (SCCs) at Week 1 pre- (-1 Wk) and postpartum (+1 Wk). **(A)** Neutrophil cell cycle reinitiation gene (*TP53*). **(B)** Cyclin-dependent kinases (*CDK1*, *CDK2*, *CDK4*). **(C)** Cyclin-dependent kinase specific-substrates (*CCNA2*, *CCNB3*, *CCND1*, *CCNE1*). **(D)** Cyclin-dependent kinase upstream gene (*CDC25A*). **(E)** Cyclin-dependent kinase downstream genes. (*DP1*, *C-Myc*) **(F)** DNA promoter-binding genes (*ORC1*, *ORC2*, *ORC4*). **(G)** Initiation factors (*MCM3*, *MCM5*, *MCM6*). The data are expressed as the mean ± SEM, n = 5 cows per group. Different superscripts (a, b, and c) represent significant differences (*P* < 0.05). Hs, health status; Wk, sampling week; Hs × Wk, interaction between health status and sampling week. *TP53*, tumor suppressor p53; *CDK1*, cyclin-dependent kinase 1; *CDK2*, cyclin-dependent kinase 2; *CDK4*, cyclin-dependent kinase 4; *CCNA2*, cyclin A2; *CCNB3*, cyclin B3; *CCND1*= cyclin D1; *CCNE1*, cyclin E1; *CDC25A*, cell division cycle 25A; *DP1*, D-prostanoid receptor 1; *C-Myc*, transcriptional regulator Myc-like; *ORC1*, origin recognition complex subunit 1; *ORC2*, origin recognition complex subunit 2; *ORC4*, origin recognition complex subunit 4; *MCM3*, minichromosome maintenance 3; *MCM5*, minichromosome maintenance 5; *MCM6*, minichromosome maintenance 6.

Compared with those in the low-SCC group, neutrophil expression levels of genes involved in the cell cycle, including *TP53*, *CDK1*, *CDK2*, *CDK4*, *CCNA2*, *CCNB3*, *CCND1*, *CCNE1*, *CDC25A*, *DP1*, *C-Myc*, *ORC1*, *ORC2*, *ORC4*, *MCM3*, *MCM5*, and *MCM6*, were significantly higher (*P <* 0.05) in high-SCC cows (**Figure 2**). *CDK2* expression was significantly higher (*P* < 0.05) in postpartum dairy cows than in prepartum cows, while the expression of other cell cycle-related genes in the neutrophils of postpartum cows was not different from that in prepartum cows (**Figure 2**). The interaction effect (Hs × Wk) was significant for *CCNA2* (*P* < 0.05).

### Formation of NETs by Neutrophils in Prepartum Cows

**Figure 3:**
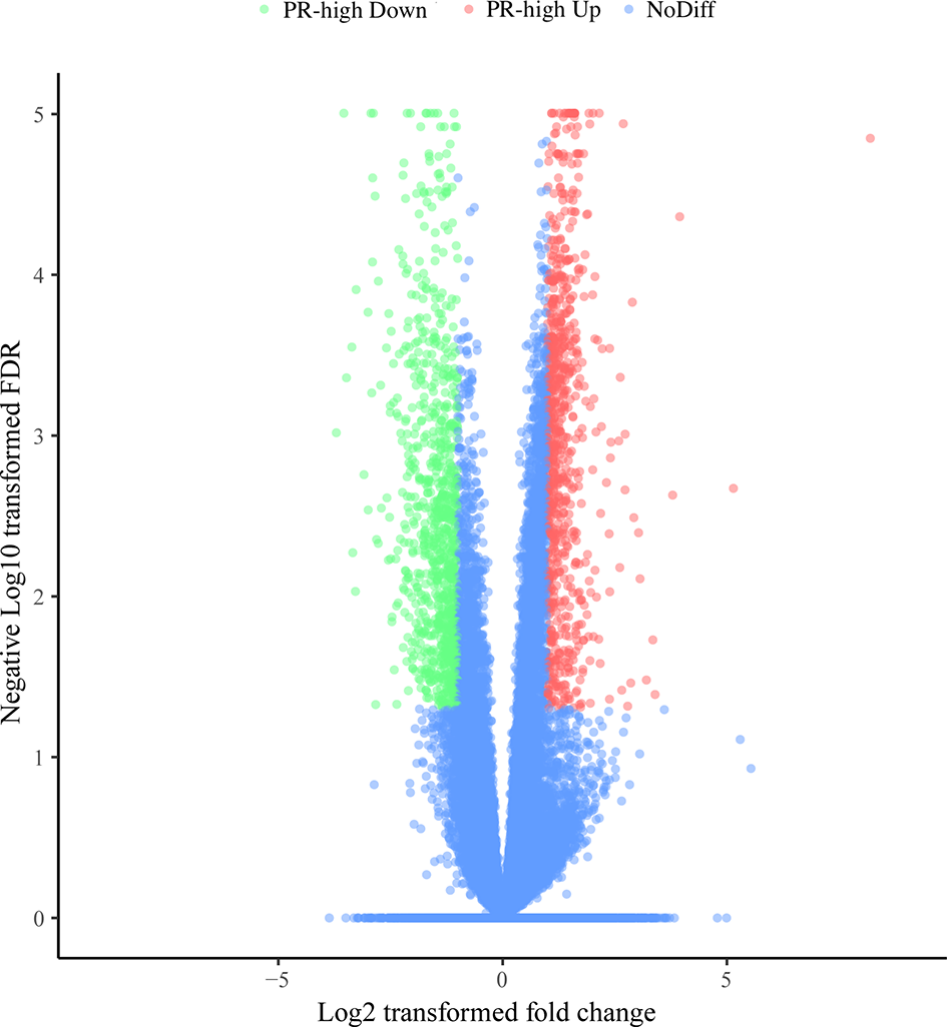
Differentially expressed genes (DEGs) in the neutrophils of dairy cows with low or high milk somatic cell counts at Week 1 prepartum. The x-axis represents the log2-fold change values of DEGs, and the y-axis indicates log10 (*P* value). The significantly up- and downregulated genes are shown as red and green dots, respectively, whereas the blue dots represent genes without significant expression changes. PR-high, prepartum high SCC.

The mRNA expression of a total of 21,648 genes was examined in the 10 cDNA libraries of blood neutrophils isolated from dairy cows (5 high-SCC cows and 5 low-SCC cows) at Week 1 prepartum, and the DEGs between the PR-low and PR-high groups are shown in **Figure 3**. A total of 1,285 DEGs (FDR < 0.05, |log2FoldChange| ≥ 1) were identified, of which 651 were downregulated and 634 were upregulated in the PR-high group compared with the PR-low group.

The GO terms enriched by the upregulated and downregulated genes are shown in **Table S3**. In total, we obtained 144, 47, and 41 upregulated GO terms in BP, CC, and MF, respectively, and 75 and 4 downregulated GO terms in BP and MF, respectively. There were more upregulated GO terms in BP, CC, and MF than downregulated GO terms. Among these, the upregulated GO terms in BP, CC, and MF were mostly related to cell cycle processes. The downregulated GO terms in BP were related to diseases, immunity, signal transduction, cell adhesion, energy metabolism, translation, and cell migration. The downregulated GO terms in MF were mainly related to immunity.

**Figure 4:**
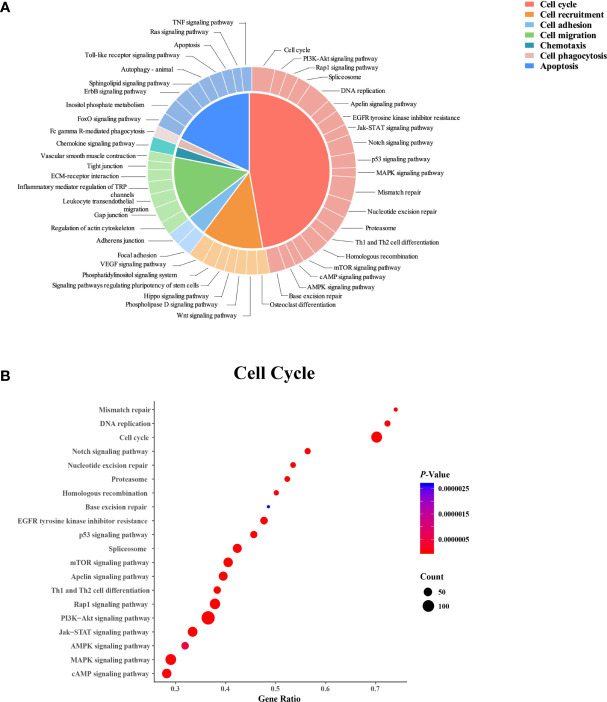
Kyoto Encyclopedia of Genes and Genomes (KEGG) pathway analysis of the differentially expressed genes in neutrophils from dairy cows with high or low somatic cell counts at Week 1 prepartum. **(A)** The KEGG pathways. **(B)** Cell cycle-related pathways. In **(B)** the x-axis shows the gene ratio, and the y-axis displays the enrichment related to the cell cycle pathway. The circle size represents the number of genes, and the color indicates the *P* value.

A total of 1,285 DEGs were mapped to 121 KEGG pathways (*P* < 0.05, **Figure 4**). Among these pathways, 47 were related to cellular functions, including the cell cycle (42.6%), apoptosis (19.2%), cell recruitment (14.9%), cell migration (14.9%), cell adhesion (4.26%), chemotaxis (2.13%), and phagocytosis (2.13%) (**Figure 4A**). The 20 pathways involved in the cell cycle are shown in **Figure 4B**.

**Figure 5:**
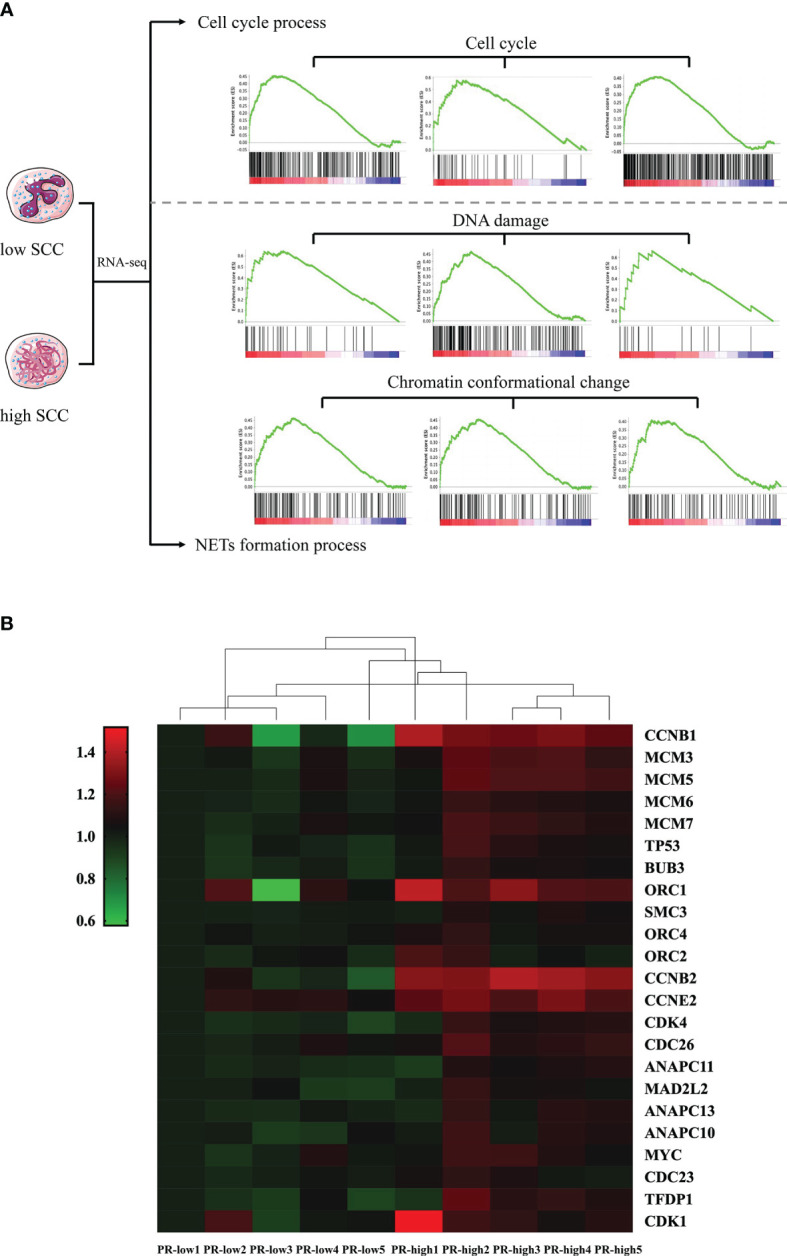
Gene set enrichment analysis (GSEA) of differentially expressed genes (DEGs) in neutrophils from dairy cows with high or low somatic cell counts at Week 1 prepartum. **(A)** The cell cycle, DNA damage, and chromatin conformational pathways identified by GSEA. **(B)** Heatmap showing hub DEGs in the cell cycle, DNA damage, and chromatin conformational pathways. PR-low, prepartum low SCC; PR-high, prepartum high SCC.

The results of GSEA are shown in **Figure 5**. These pathways (|NES| > 1, P < 0.05, FDR < 0.05) were related to the cell cycle, DNA damage, and chromatin conformation (**Figure 5A**), and the hub genes involved in these selected pathways included *TP53*, CDKs, CCNs, DP1, ORCs, and MCMs (**Figure 5B**).

## Discussion

To our knowledge, this work was one of a few studies using RNA-seq analysis to investigate the relationship between functional changes in neutrophils and mastitis risk in transition dairy cows. We found that the transcriptome changes in blood neutrophils during the transition period were mainly associated with cell cycle-related processes. However, neutrophils in the peripheral blood are terminally differentiated cells that do not proliferate, and the reactivation of the cell cycle that was observed in this study is similar to NETosis. These findings suggested that prepartum formation of blood NETs increases the postpartum mastitis risk of dairy cows.

The functions of neutrophils in immunity and microbe elimination are affected by pathological conditions (38). Our previous work showed that the serum ROS concentrations of dairy cows with low and high SCCs changed differently after calving (8). This difference indicated that changes in ROS enhance the formation of NETs in dairy cows with high mastitis risk. Indeed, in the present study, analysis of serum NET-related indices confirmed the difference in the formation of NETs in cows with low and high SCCs at Week 1 pre- and postpartum. Among these indices, DNase I is a NET marker that can directly reflect the degradation of NETs *in vivo* (39). Both MPO and NE are the main components by which NETs exert their antimicrobial effects (40). IL-1β and IL-8 are external inducers that stimulate neutrophils to form NETs (41), and ROS stimulate neutrophils to directly generate NETs and promote their release (12). 8-OHdG reflects the oxidative damage of DNA (42, 43), which is necessary for neutrophils to generate NETs (44). In this study, neutrophils in dairy cows with high mastitis risk may be stimulated by IL-1β or IL-8 in the blood, and the DNA conformation in the nucleus might be damaged by high ROS levels. Then, NETs formed in these cows by the binding of DNA to cytoplasmic granules and were ultimately released into the blood.

The formation of NETs is beneficial to cow health. However, excessive formation of NETs, in turn, can damage tissues and organs, resulting in increased neutrophil accumulation in inflamed tissues and organs (12). The MDA and PC concentrations reflect oxidative damage of lipids and proteins, respectively (43). The results in the present study revealed that dairy cows with high SCCs had higher levels of MDA and PC resulting from the excessive formation of NETs. IL-17 can mediate the activation of neutrophil mobilization, effectively mediating the inflammatory response in tissue (45). In the present study, an increased IL-17 concentration was observed in cows with high mastitis risk, indicating that NETs in the blood may increase the release of neutrophils into blood and milk. Taken together, these results indicated that the formation of blood NETs increased the mastitis risk of dairy cows during the transition period. Consistently, our previous studies (8) also suggested that alterations in serum TNF-α, IL-6, and PSGL-1 concentrations between cows with low and high SCCs were associated with changes in neutrophil chemotaxis, adhesion, and apoptosis. In addition, the neutrophil counts and its functional factors tended to be higher in dairy cows with high SCCs than those with low SCCs from prepartum to postpartum in both our previous (8) and present studies. Thus, we suggest that the functional factors of neutrophils with different SCCs might be potential indicators for mastitis risk from late gestation to early lactation. However, the behind mechanism warrants further study.

Transcriptomic analysis revealed that the changes in neutrophil functions in dairy cows with different SCCs were mainly associated with immunity- and cell cycle-related processes. In this study, we focused on the formation of NETs because of its capacity of directly aggravating mastitis risk. However, the pathways involved in NETosis are poorly understood. In neutrophils, cell-cycle pathways are repurposed for controlling NETosis (15, 46). Before cell cycle processes are activated, gene products of ORC*s* (*ORC1*, *ORC2*, and *ORC4*) bind to the DNA start site on the chromosome and other transcription initiation factors, such as the MCM family (*MCM3*, *MCM5*, and *MCM6*), to form a prereplication complex, shifting cells from the G0 phase to the G1 phase (47). In this study, the increased gene expression of *ORC1*, *ORC2*, *ORC4*, *MCM3*, *MCM5*, and *MCM6* in the neutrophils of cows with high SCCs indicated that the cell cycle may be activated in neutrophils in high mastitis risk cows during the transition period. However, it is still unclear how the cell cycle is activated because blood neutrophils are terminal cells and cannot re-enter the cell cycle alone. *TP53* is a critical transcription factor that regulates the expression levels of cyclin-dependent kinases (*CDK2* and *CDK4*) and their specific substrates (*CCNE1* and *CCND1*) to initiate G1 phase (48–50). During G1 phase activation, *CDC25A* regulates *CDKs* (*CDK2* and *CDK1*) and their specific substrates (*CCNA2* and *CCNB3*) to start the S phase, G2 phase, and M phase (51). Increased gene expression of *TP53*, *CDK1*, *CDK2*, *CDK4*, *CCNA2*, *CCNB3*, *CCND1*, *CCNE1*, and *CDC25A* was observed in our study and suggested that neutrophil mitosis might occur in dairy cows with high SCCs. The processes of chromosome unwinding during mitosis highly correspond to NETosis. The cell-cycle kinases *CDK4* can mediate the formation of NETs (15). We suggest that neutrophils in dairy cows with high mastitis risk may not actually reactivate the cell cycle but do undergo NETosis.

After neutrophils are stimulated, their chromatin is depolymerized, the nuclear membrane is ruptured, and the cell membrane eventually breaks, releasing NETs (12, 13). NETosis is a new type of neutrophil cell death that is distinct from apoptosis or necrosis. NETosis is an active process characterized by the internal breakdown of nuclear membranes (15). The expression level of *DP1* and *c-Myc* in the nucleus is increased by the activation of the cell cycle and the coregulation of *CDKs*, leading to nuclear membrane rupture (52). In this study, the high expression of *DP1* and *C-Myc* in the neutrophils of high mastitis risk cows revealed that the formation of blood NETs increased the mastitis risk of dairy cows during the transition period. Mastitis leads to great economic losses in the dairy industry and impairs animal health. A prepartum prognosis of mastitis is crucial in transition dairy cows (53). No significant difference was found in the expression of cell cycle pathway genes between the pre- and postpartum dairy cows in the present study, indicating that the formation of NETs observed in postpartum dairy cows occurred during the prepartum period. Therefore, we further investigated whether prepartum functional changes in neutrophils were consistent with the changes in neutrophils during the transition period.

Consistent with the functional changes in neutrophils in transition dairy cows, GO term analysis of DEGs in the neutrophils of cows with high or low SCCs at Week 1 prepartum also showed changes related to the cell cycle. Among the cellular pathways related to the functional variations in neutrophils, the proportion of cell cycle-related pathways was the largest (42.6%). The other pathways were related to cell recruitment, adhesion, migration, chemotaxis, phagocytosis, and apoptosis. Decreases in neutrophil recruitment, adhesion, migration, chemotaxis, phagocytosis, and apoptosis were observed in dairy cows with diseases (6). DNA damage can reactivate the cell cycle and induce chromatin conformation changes, which is closely related to the formation of NETs (15). The present study showed that pathways related to the cell cycle, DNA damage, and chromosomal conformation in neutrophils were different between PR-low and PR-high cows. The hub genes of these pathways, such as *CDK4*, were involved in the cell cycle and were related to NETosis (15). The expression of the hub genes was largely confirmed by qPCR, and the analysis of serum NET-related indices further confirmed the formation of NETs in high-SCC dairy cows at Week 1 prepartum. The analysis of prepartum neutrophil transcriptome differences indicated that the formation of NETs by blood neutrophils during the prepartum period was closely linked with the increased SCCs in postpartum milk. When blood neutrophils reach the inflamed mammary gland, they may generate and release NETs and continuously kill bacteria. However, excessive NET accumulation could damage the mammary gland, causing mammary epithelial cells to slough off and more neutrophils to enter the inflamed mammary gland. Finally, the excessive generation of NETs led to increased SCCs and negatively impacted milk production and milk quality. The abnormal state of neutrophils persisted during the transition period, leading to the differences in SCCs in postpartum milk observed in the two groups of dairy cows.

## Conclusions

**Figure 6:**
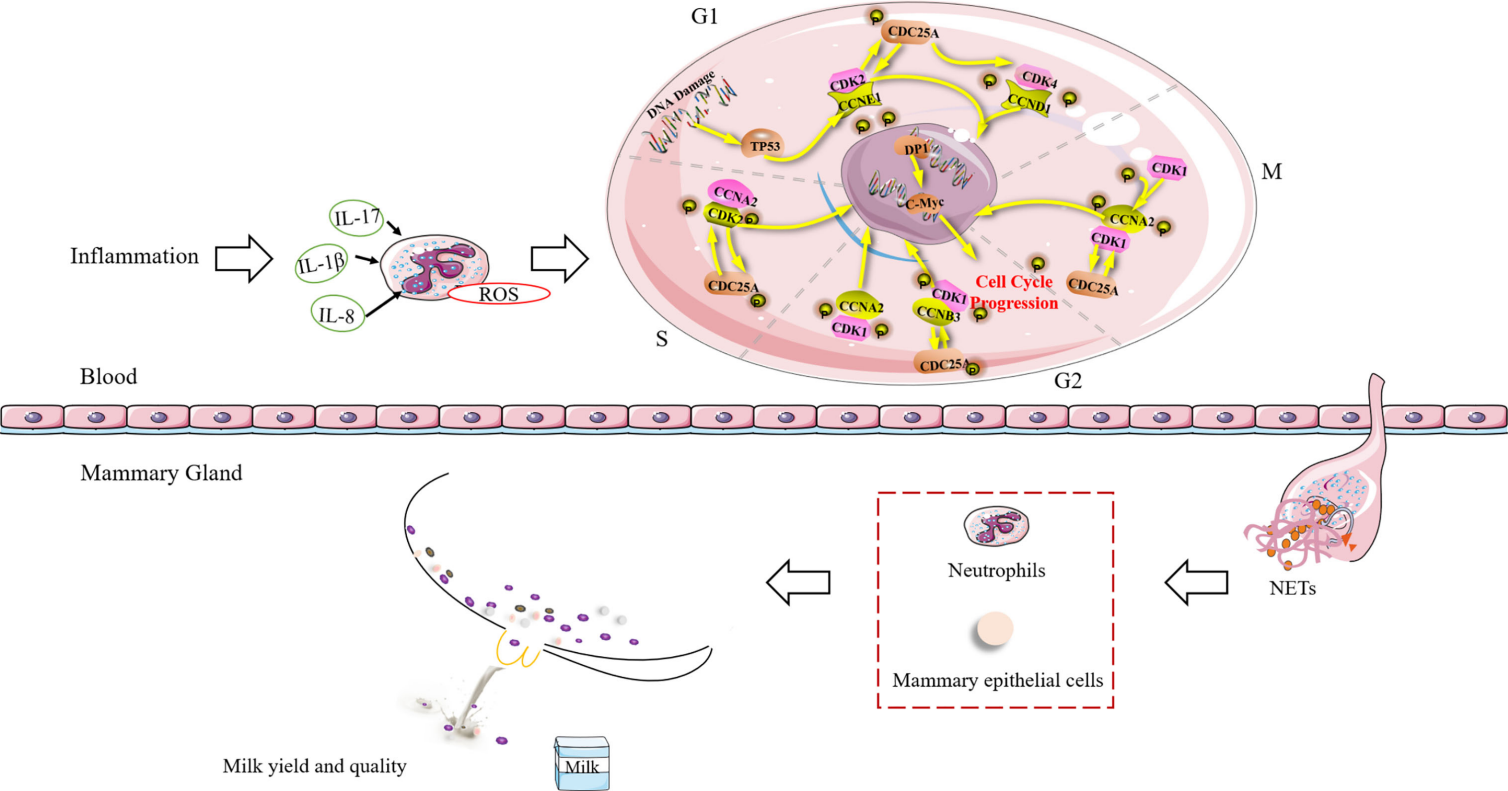
Schematic diagram of the relationship between NET formation in blood and the risk of mastitis in dairy cows during the transition period. *TP53*, Tumor suppressor p53; *CCNA2*, Cyclin A2; *CCNB3*, Cyclin B3; *CCND1*= Cyclin D1; *CCNE1*, Cyclin E1; *CDC25A*, Cell division cycle 25A; *CDK1*, Cyclin-dependent kinase 1; *CDK2*, Cyclin-dependent kinase 2; *CDK4*, Cyclin-dependent kinase 4; *DP1*, D-prostanoid receptor 1; G1, G2, M, and S, different stages of cellular differentiation; IL, interleukin; NETs, neutrophil extracellular traps; ROS, reactive oxygen species.

Our study showed that the cell cycle of blood neutrophils is highly associated with milk SCCs in transition dairy cows. The chromosome of neutrophils, which are terminal cells, unwinds and binds to cytoplasmic granular proteins to form and release NETs, but these cells do not enter the cell cycle. After neutrophils migrate into the mammary gland and enter into NETosis, NETs continue to exert their antimicrobial effects. However, the excessive accumulation of NETs might recruit more neutrophils to the mammary gland, resulting in increased SCCs and reduced milk production and milk quality. The NETs formed in dairy cows before calving are present during the transition period, which might explain the variations in SCCs. In conclusion, the formation of blood NETs in transition dairy cows can increase the risk of postpartum mastitis (**Figure 6**).

## Data Availability Statement

The data used to support the findings of this study are included within the article. The original RNA-Seq datasets have been deposited in the NCBI SRA database and can be accessed using accession number PRJNA752262.

## Ethics Statement

The animal study was reviewed and approved by the Institutional Animal Care and Use Committee of Zhejiang University.

## Author Contributions

R-WG and L-YJ performed the experiments. L-YJ analyzed the data and wrote the original manuscript. J-XL, FS, and F-QZ obtained the funding, contributed to the study design, and revised the manuscript. H-ZS revised the manuscript. All authors reviewed the final manuscript.

## Funding

This study was financially supported by grants from the China-USA Intergovernmental Collaborative Project in S & T Innovation under the National Key R & D Program (No. 2018YFE0111700, Beijing).

## Conflict of Interest

The authors declare that the research was conducted in the absence of any commercial or financial relationships that could be construed as a potential conflict of interest.

## Publisher’s Note

All claims expressed in this article are solely those of the authors and do not necessarily represent those of their affiliated organizations, or those of the publisher, the editors and the reviewers. Any product that may be evaluated in this article, or claim that may be made by its manufacturer, is not guaranteed or endorsed by the publisher.

## References

[1] Miles AM Huson HJ. Time- and Population-Dependent Genetic Patterns Underlie Bovine Milk Somatic Cell Count. J Dairy Sci (2020) 103(9):8292–304. doi: 10.3168/jds.2020-18322 32622601

[2] Horst EA Kvidera SK Baumgard LH. Invited Review: The Influence of Immune Activation on Transition Cow Health and Performance-A Critical Evaluation of Traditional Dogmas. J Dairy Sci (2021) 104(8):8380–410. doi: 10.3168/jds.2021-20330 34053763

[3] Gröhn YT Wilson DJ González RN Hertl JA Schulte H Bennett G. Effect of Pathogen-Specific Clinical Mastitis on Milk Yield in Dairy Cows. J Dairy Sci (2004) 87(10):3358–74. doi: 10.3168/jds.S0022-0302(04)73472-4 15377615

[4] Souza FN Blagitz MG Batista CF Takano PV Gargano RG Diniz SA. Immune Response in Nonspecific Mastitis: What Can It Tell Us? J Dairy Sci (2020) 103(6):5376–86. doi: 10.3168/jds.2019-17022 32229113

[5] Gross JJ Grossen-Rösti L Wall SK Wellnitz O Bruckmaier RM. Metabolic Status Is Associated With the Recovery of Milk Somatic Cell Count and Milk Secretion After Lipopolysaccharide-Induced Mastitis in Dairy Cows. J Dairy Sci (2020) 103(6):5604–15. doi: 10.3168/jds.2019-18032 32253039

[6] Bassel LL Caswell JL. Bovine Neutrophils in Health and Disease. Cell Tissue Res (2018) 371(3):617–37. doi: 10.1007/s00441-018-2789-y 29445861

[7] Bobbo T Penasa M Cassandro M. Short Communication: Genetic Aspects of Milk Differential Somatic Cell Count in Holstein Cows: A Preliminary Analysis. J Dairy Sci (2019) 102(5):4275–9. doi: 10.3168/jds.2018-16092 30827547

[8] Guan RW Wang DM Wang BB Jiang LY Liu JX. Prognostic Potential of Pre-Partum Blood Biochemical and Immune Variables for Postpartum Mastitis Risk in Dairy Cows. BMC Vet Res (2020) 16(1):136. doi: 10.1186/s12917-020-02314-6 32408873 PMC7222453

[9] Soehnlein O Steffens S Hidalgo A Weber C. Neutrophils as Protagonists and Targets in Chronic Inflammation. Nat Rev Immunol (2017) 17(4):248–61. doi: 10.1038/nri.2017.10 28287106

[10] Ju Z Jiang Q Wang J Wang X Yang C Sun Y. Genome-Wide Methylation and Transcriptome of Blood Neutrophils Reveal the Roles of DNA Methylation in Affecting Transcription of Protein-Coding Genes and Mirnas in E Coli-Infected Mastitis Cows. BMC Genomics (2020) 21(1):102. doi: 10.1186/s12864-020-6526-z 32000686 PMC6993440

[11] Van Rees DJ Szilagyi K Kuijpers TW Matlung HL Van Den Berg TK. Immunoreceptors on Neutrophils. Semin Immunol (2016) 28(2):94–108. doi: 10.1016/j.smim.2016.02.004 26976825 PMC7129252

[12] Papayannopoulos V. Neutrophil Extracellular Traps in Immunity and Disease. Nat Rev Immunol (2018) 18(2):134–47. doi: 10.1038/nri.2017.105 28990587

[13] Fuchs TA Abed U Goosmann C Hurwitz R Schulze I Wahn V. Novel Cell Death Program Leads to Neutrophil Extracellular Traps. J Cell Biol (2007) 176(2):231–41. doi: 10.1083/jcb.200606027 PMC2063942 17210947

[14] Yuen J Pluthero FG Douda DN Riedl M Cherry A Ulanova M. NETosing Neutrophils Activate Complement Both on Their Own NETs and Bacteria *via* Alternative and Non-Alternative Pathways. Front Immunol (2016) 7:137. doi: 10.3389/fimmu.2016.00137 27148258 PMC4831636

[15] Amulic B Knackstedt SL Abu Abed U Deigendesch N Harbort CJ Caffrey BE. Cell-Cycle Proteins Control Production of Neutrophil Extracellular Traps. Dev Cell (2017) 43(4):449–62.e5. doi: 10.1016/j.devcel.2017.10.013 29103955

[16] Alhussien M Manjari P Mohammed S Sheikh AA Reddi S Dixit S. Incidence of Mastitis and Activity of Milk Neutrophils in Tharparkar Cows Reared Under Semi-Arid Conditions. Trop Anim Health Prod (2016) 48(6):1291–5. doi: 10.1007/s11250-016-1068-8 27154217

[17] Dey SS Kester L Spanjaard B Bienko M van Oudenaarden A. Integrated Genome and Transcriptome Sequencing of the Same Cell. Nat Biotechnol (2015) 33(3):285–9. doi: 10.1038/nbt.3129 PMC4374170 25599178

[18] Crookenden MA Moyes KM Kuhn-Sherlock B Lehnert K Walker CG Loor JJ. Transcriptomic Analysis of Circulating Neutrophils in Metabolically Stressed Peripartal Grazing Dairy Cows. J Dairy Sci (2019) 102(8):7408–20. doi: 10.3168/jds.2019-16367 31178180

[19] National Research Council. Nutrient Requirements of Dairy Cattle: Seventh Revised Edition, 2001. Washington, DC: The National Academies Press (2001). Available at: https://nap.nationalacademies.org/catalog/9825/nutrient-requirements-of-dairy-cattle-seventh-revised-edition-2001

[20] Freitas M Porto G Lima JL Fernandes E. Isolation and Activation of Human Neutrophils in Vitro. The Importance of the Anticoagulant Used During Blood Collection. Clin Biochem (2008) 41(7-8):570–5. doi: 10.1016/j.clinbiochem.2007.12.021 18226596

[21] Feng S Yu H Yu Y Geng Y Li D Yang C. Levels of Inflammatory Cytokines IL-1β, IL-6, IL-8, IL-17a, and TNF-α in Aqueous Humour of Patients With Diabetic Retinopathy. J Diabetes Res (2018) 2018:8546423. doi: 10.1155/2018/8546423 29850610 PMC5904804

[22] Sang H Zhang L Li J. Anti-Benzopyrene-7,8-Diol-9,10-Epoxide Induces Apoptosis *via* Mitochondrial Pathway in Human Bronchiolar Epithelium Cells Independent of the Mitochondria Permeability Transition Pore. Food Chem Toxicol (2012) 50(7):2417–23. doi: 10.1016/j.fct.2012.04.041 22565279

[23] Del Rio D Stewart AJ Pellegrini N. A Review of Recent Studies on Malondialdehyde as Toxic Molecule and Biological Marker of Oxidative Stress. Nutr Metab Cardiovasc Dis (2005) 15(4):316–28. doi: 10.1016/j.numecd.2005.05.003 16054557

[24] Wei QY Chen WF Zhou B Yang L Liu ZL. Inhibition of Lipid Peroxidation and Protein Oxidation in Rat Liver Mitochondria by Curcumin and Its Analogues. Biochim Biophys Acta (2006) 1760(1):70–7. doi: 10.1016/j.bbagen.2005.09.008 16236451

[25] Valavanidis A Vlachogianni T Fiotakis C. 8-Hydroxy-2’ -Deoxyguanosine (8-Ohdg): A Critical Biomarker of Oxidative Stress and Carcinogenesis. J Environ Sci Health C Environ Carcinog Ecotoxicol Rev (2009) 27(2):120–39. doi: 10.1080/10590500902885684 19412858

[26] Brinkmann V Laube B Abu Abed U Goosmann C Zychlinsky A. Neutrophil Extracellular Traps: How to Generate and Visualize Them. J Vis Exp (2010) (36):e1724. doi: 10.3791/1724 PMC3125121 20182410

[27] Caudrillier A Kessenbrock K Gilliss BM Nguyen JX Marques MB Monestier M. Platelets Induce Neutrophil Extracellular Traps in Transfusion-Related Acute Lung Injury. J Clin Invest (2012) 122(7):2661–71. doi: 10.1172/jci61303 PMC3386815 22684106

[28] Middleton EA He XY Denorme F Campbell RA Ng D Salvatore SP. Neutrophil Extracellular Traps Contribute to Immunothrombosis in Covid-19 Acute Respiratory Distress Syndrome. Blood (2020) 136(10):1169–79. doi: 10.1182/blood.2020007008 PMC7472714 32597954

[29] Thiyagarajan D Rekvig OP Seredkina N. Tnfα Amplifies Dnasei Expression in Renal Tubular Cells While IL-1β Promotes Nuclear Dnasei Translocation in an Endonuclease-Inactive Form. PloS One (2015) 10(6):e0129485. doi: 10.1371/journal.pone.0129485 26065428 PMC4465975

[30] Tokuhiro T Ishikawa A Sato H Takita S Yoshikawa A Anzai R. Oxidized Phospholipids and Neutrophil Elastase Coordinately Play Critical Roles in NET Formation. Front Cell Dev Biol (2021) 9:718586. doi: 10.3389/fcell.2021.718586 34568331 PMC8458647

[31] Mohamed JS Lopez MA Boriek AM. Mechanical Stretch Up-Regulates Microrna-26a and Induces Human Airway Smooth Muscle Hypertrophy by Suppressing Glycogen Synthase Kinase-3β. J Biol Chem (2010) 285(38):29336–47. doi: 10.1074/jbc.M110.101147 PMC2937966 20525681

[32] Patch AM Nones K Kazakoff SH Newell F Wood S Leonard C. Germline and Somatic Variant Identification Using Bgiseq-500 and Hiseq X Ten Whole Genome Sequencing. PloS One (2018) 13(1):e0190264. doi: 10.1371/journal.pone.0190264 29320538 PMC5761881

[33] Patro R Duggal G Love MI Irizarry RA Kingsford C. Salmon Provides Fast and Bias-Aware Quantification of Transcript Expression. Nat Methods (2017) 14(4):417–9. doi: 10.1038/nmeth.4197 PMC5600148 28263959

[34] Langfelder P Horvath S. Wgcna: An R Package for Weighted Correlation Network Analysis. BMC Bioinf (2008) 9:559. doi: 10.1186/1471-2105-9-559 PMC2631488 19114008

[35] Taylor SC Nadeau K Abbasi M Lachance C Nguyen M Fenrich J. The Ultimate Qpcr Experiment: Producing Publication Quality, Reproducible Data the First Time. Trends Biotechnol (2019) 37(7):761–74. doi: 10.1016/j.tibtech.2018.12.002 30654913

[36] Love MI Huber W Anders S. Moderated Estimation of Fold Change and Dispersion for RNA-Seq Data With Deseq2. Genome Biol (2014) 15(12):550. doi: 10.1186/s13059-014-0550-8 25516281 PMC4302049

[37] Subramanian A Tamayo P Mootha VK Mukherjee S Ebert BL Gillette MA. Gene Set Enrichment Analysis: A Knowledge-Based Approach for Interpreting Genome-Wide Expression Profiles. Proc Natl Acad Sci USA (2005) 102(43):15545–50. doi: 10.1073/pnas.0506580102 PMC1239896 16199517

[38] Amulic B Cazalet C Hayes GL Metzler KD Zychlinsky A. Neutrophil Function: From Mechanisms to Disease. Annu Rev Immunol (2012) 30:459–89. doi: 10.1146/annurev-immunol-020711-074942 22224774

[39] Mallavia B Liu F Lefrançais E Cleary SJ Kwaan N Tian JJ. Mitochondrial DNA Stimulates TLR9-Dependent Neutrophil Extracellular Trap Formation in Primary Graft Dysfunction. Am J Respir Cell Mol Biol (2020) 62(3):364–72. doi: 10.1165/rcmb.2019-0140OC PMC7055700 31647878

[40] Muñoz-Caro T Lendner M Daugschies A Hermosilla C Taubert A. NADPH Oxidase, MPO, NE, ERK1/2, P38 MAPK and Ca^2+^ Influx Are Essential for Cryptosporidium Parvum-Induced NET Formation. Dev Comp Immunol (2015) 52(2):245–54. doi: 10.1016/j.dci.2015.05.007 26026247

[41] Mistry P Carmona-Rivera C Ombrello AK Hoffmann P Seto NL Jones A. Dysregulated Neutrophil Responses and Neutrophil Extracellular Trap Formation and Degradation in PAPA Syndrome. Ann Rheum Dis (2018) 77(12):1825–33. doi: 10.1136/annrheumdis-2018-213746 PMC6728908 30131320

[42] Köroğlu KM Çevik Ö Şener G Ercan F. Apocynin Alleviates Cisplatin-Induced Testicular Cytotoxicity by Regulating Oxidative Stress and Apoptosis in Rats. Andrologia (2019) 51(4):e13227. doi: 10.1111/and.13227 30623469

[43] Matzi V Greilberger JF Lindenmann J Neuboeck N Nuhsbaumer S Zelzer S. Application of Hyperbaric Oxygen Reduce Oxidative Damage of Plasmatic Carbonyl Proteins and 8-OHdG by Activating Glutathion Peroxidase. Clin Lab (2015) 61(5-6):587–93. doi: 10.7754/clin.lab.2014.140929 26118193

[44] Boettcher M Eschenburg G Mietzsch S Jiménez-Alcázar M Klinke M Vincent D. Therapeutic Targeting of Extracellular DNA Improves the Outcome of Intestinal Ischemic Reperfusion Injury in Neonatal Rats. Sci Rep (2017) 7(1):15377. doi: 10.1038/s41598-017-15807-6 29133856 PMC5684414

[45] Frangou E Chrysanthopoulou A Mitsios A Kambas K Arelaki S Angelidou I. REDD1/Autophagy Pathway Promotes Thromboinflammation and Fibrosis in Human Systemic Lupus Erythematosus (SLE) Through NETs Decorated With Tissue Factor (TF) and Interleukin-17a (IL-17a). Ann Rheum Dis (2019) 78(2):238–48. doi: 10.1136/annrheumdis-2018-213181 PMC6352428 30563869

[46] Thiam HR Wong SL Wagner DD Waterman CM. Cellular Mechanisms of NETosis. Annu Rev Cell Dev Biol (2020) 36:191–218. doi: 10.1146/annurev-cellbio-020520-111016 32663035 PMC8499668

[47] Sun W Hola M Pedley K Tada S Blow JJ Todorov IT. The Replication Capacity of Intact Mammalian Nuclei in Xenopus Egg Extracts Declines With Quiescence, But the Residual DNA Synthesis Is Independent of Xenopus Mcm Proteins. J Cell Sci (2000) 113(Pt 4):683–95. doi: 10.1242/jcs.113.4.683 10652261

[48] Mariño G Niso-Santano M Baehrecke EH Kroemer G. Self-Consumption: The Interplay of Autophagy and Apoptosis. Nat Rev Mol Cell Biol (2014) 15(2):81–94. doi: 10.1038/nrm3735 24401948 PMC3970201

[49] Kruiswijk F Labuschagne CF Vousden KH. P53 in Survival, Death and Metabolic Health: A Lifeguard With a Licence to Kill. Nat Rev Mol Cell Biol (2015) 16(7):393–405. doi: 10.1038/nrm4007 26122615

[50] Shin DY Sung Kang H Kim GY Kim WJ Yoo YH Choi YH. Decitabine, a DNA Methyltransferases Inhibitor, Induces Cell Cycle Arrest at G2/M Phase Through P53-Independent Pathway in Human Cancer Cells. BioMed Pharmacother (2013) 67(4):305–11. doi: 10.1016/j.biopha.2013.01.004 23582784

[51] Jabbour-Leung NA Chen X Bui T Jiang Y Yang D Vijayaraghavan S. Sequential Combination Therapy of CDK Inhibition and Doxorubicin Is Synthetically Lethal in P53-Mutant Triple-Negative Breast Cancer. Mol Cancer Ther (2016) 15(4):593–607. doi: 10.1158/1535-7163.Mct-15-0519 26826118 PMC4873336

[52] Zhang J Li H Yabut O Fitzpatrick H D’Arcangelo G Herrup K. Cdk5 Suppresses the Neuronal Cell Cycle by Disrupting the E2F1-DP1 Complex. J Neurosci (2010) 30(15):5219–28. doi: 10.1523/jneurosci.5628-09.2010 PMC2862267 20392944

[53] Hailemariam D Mandal R Saleem F Dunn SM Wishart DS Ametaj BN. Identification of Predictive Biomarkers of Disease State in Transition Dairy Cows. J Dairy Sci (2014) 97(5):2680–93. doi: 10.3168/jds.2013-6803 24630653

