# Supplementary material for: Formation of Blood Neutrophil Extracellular Traps Increases the Mastitis Risk of Dairy Cows During the Transition Period
Source: Front Immunol. 2022 Apr 27;13:880578. doi: 10.3389/fimmu.2022.880578 (PMC9092530; doi:10.3389/fimmu.2022.880578)
Supplement: Supplementary file 1 [file DataSheet_1.docx]

**Table S1.** Primer sequences used in real-time PCR assay.

| Gene^1^ | GeneBank ID^2^ | Primer sequence^3^ | Length |
| --- | --- | --- | --- |
| β-actin | NM_173979.3 | GTCGACACCGCAACCAGT | 244 |
|  |  | CCGTGCTCAATGGGGTACTT |  |
| TP53 | NM_174201.2 | GCTCACTCTAGCCACCTGAA | 223 |
|  |  | AGACTCCCAGGGGGTTTCTA |  |
| CDK1 | NM_174016.2 | TTGGCAGATTTTGGCCTTGC | 366 |
|  |  | CGAGAGCAGATCCAAGCCAT |  |
| CDK2 | NM_001014934.1 | CACTCAGCTGCCCCTCTTAG | 153 |
|  |  | CCAGCCCTCTTAAGCAACGA |  |
| CDK4 | NM_001037594.2 | ACACAAGCGAATCTCTGCCT | 167 |
|  |  | TTGCCTTCTCATTGGTGGGG |  |
| CCNA2 | NM_001075123.1 | AACTGCAGAACGAGACCCTG | 100 |
|  |  | GCAGTGCCCACAAGTTGAAG |  |
| CCNB3 | XM_024988519.1 | GATCAGGGGGTAGTGTCCCT | 288 |
|  |  | TGGGTTGGTGAAGGTTGCTT |  |
| CCND1 | NM_001046273.2 | GCTTCCTCTCCTATCACCGC | 224 |
|  |  | TCAGATGTTCACGTCACGCA |  |
| CCNE1 | NM_001192776.1 | ACCGATGTCTCTGTTCGCTC | 152 |
|  |  | CAGGGGTTTTCACAGGCTCT |  |
| CDC25A | NM_001101100.2 | TGTACAGCCGCCTGAAGAAG | 222 |
|  |  | GACGTCTTCCAAGGACGGAG |  |
| DP1 | NM_001076029.1 | CCAGAACCAGCCTTCAGACC | 216 |
|  |  | TGATCGTAGGCCGACTCACT |  |
| C-Myc | NM_001046074.2 | CAGCGTACATCCTGTCGGTC | 154 |
|  |  | GGGAGTCCAGTTCCTCCCTC |  |
| ORC1 | NM_001014918.1 | TTGCAGAGTTCCGTCGATCAG | 250 |
|  |  | TGAAGCCCTTCACTCCTCTTCA |  |
| ORC2 | NM_001101942.2 | TCGTCACCTAGAACCGGGAT | 246 |
|  |  | TCACACTCTGGAACAAGCACA |  |
| ORC4 | XM_005202444.4 | TGGAGTTTCAGGCAGACTTCTC | 156 |
|  |  | TCGTATGATTCAAAGGCAAGGC |  |
| MCM3 | NM_001013586.2 | AGCCTTTGCCTTCATTCCCT | 220 |
|  |  | ATGGTGTGGCTTGTTTGGGA |  |
| MCM5 | NM_001075290.2 | ATGCAGCGCAAGGTCCTCTA | 151 |
|  |  | CACATTTCCTGGATCTGCAGCC |  |
| MCM6 | NM_001046234.1 | AGCTGGATTGAAAGGCTCCAC | 159 |
|  |  | AGCAGAACTCCATCCAGACTC |  |

^1^TP53 = Tumor suppressor p53; CDK1 = Cyclin-dependent kinase 1; CDK2 = Cyclin-dependent kinase 2; CDK4 = Cyclin-dependent kinase 4; CCNA2 = Cyclin A2; CCNB3 = Cyclin B3; CCND1= CyclinD1; CCNE1 = CyclinE1; CDC25A = Cell division cycle 25A; DP1 = D-prostanoid receptor 1; ORC1 = Origin recognition complex subunit 1; ORC2 = Origin recognition complex subunit 2; ORC4 = Origin recognition complex subunit 4; MCM3 = Minichromosome maintenance 3; MCM5 = Minichromosome maintenance 5; MCM6 = Minichromosome maintenance 6.

^2^ GenBank Accession Number.

^3^ Shown as forward primer followed by reverse primer.

**Table S2**. Raw data and clean data generated by RNA sequencing of neutrophils from dairy cows with high or low somatic cell counts (SCCs) at Week 1 pre- and postpartum.

| Sample^1^ | Raw reads ×10^6^ | Raw bases ×10^9^ | Clean reads ×10^6^ | Clean bases ×10^9^ | Error rate (%) | Q20 (%) | Q30 (%) | GC content (%) | Total mapping (%) |
| --- | --- | --- | --- | --- | --- | --- | --- | --- | --- |
| PR-low1 | 44.4 | 6.22 | 39.7 | 6.22 | 10.6 | 95.9 | 90.9 | 47.0 | 89.5 |
| PR-low2 | 47.0 | 6.61 | 42.5 | 6.60 | 9.57 | 97.0 | 93.2 | 49.0 | 90.5 |
| PR-low3 | 44.4 | 6.16 | 40.4 | 6.16 | 9.01 | 95.5 | 90.0 | 49.0 | 90.1 |
| PR-low4 | 45.7 | 6.38 | 41.1 | 6.37 | 10.1 | 96.1 | 91.3 | 50.0 | 89.8 |
| PR-low5 | 42.8 | 5.93 | 38.6 | 5.93 | 9.81 | 95.9 | 90.7 | 50.0 | 90.3 |
| PP-low1 | 45.7 | 6.40 | 41.0 | 6.40 | 10.3 | 96.0 | 91.0 | 49.0 | 89.9 |
| PP-low2 | 46.4 | 6.55 | 42.2 | 6.55 | 9.05 | 96.9 | 93.0 | 49.0 | 90.9 |
| PP-low3 | 45.9 | 6.44 | 41.4 | 6.44 | 9.80 | 95.9 | 90.7 | 48.0 | 90.3 |
| PP-low4 | 46.9 | 6.55 | 42.2 | 6.55 | 10.0 | 96.0 | 90.9 | 51.0 | 89.9 |
| PP-low5 | 44.7 | 6.22 | 40.2 | 6.22 | 10.1 | 95.4 | 89.7 | 50.0 | 90.0 |
| PR-high1 | 44.1 | 6.38 | 40.1 | 6.38 | 9.07 | 96.9 | 93.0 | 41.0 | 90.8 |
| PR-high2 | 46.0 | 6.22 | 40.7 | 6.22 | 11.5 | 96.3 | 91.9 | 47.0 | 88.6 |
| PR-high3 | 46.4 | 6.45 | 41.4 | 6.45 | 10.8 | 95.7 | 90.4 | 50.0 | 89.1 |
| PR-high4 | 44.4 | 6.14 | 40.3 | 6.14 | 9.23 | 96.1 | 91.0 | 50.0 | 90.6 |
| PR-high5 | 47.6 | 6.50 | 42.3 | 6.49 | 11.1 | 95.8 | 90.7 | 50.0 | 89.0 |
| PP-high1 | 47.2 | 6.61 | 41.7 | 6,61 | 11.7 | 96.8 | 92.9 | 47.0 | 88.3 |
| PP-high2 | 51.9 | 7.28 | 45.8 | 7.28 | 11.8 | 96.6 | 92.8 | 47.0 | 88.1 |
| PP-high3 | 46.1 | 6.51 | 41.0 | 6.51 | 11.1 | 95.5 | 90.1 | 47.0 | 88.9 |
| PP-high4 | 48.2 | 6.80 | 43.3 | 6.80 | 10.2 | 96.0 | 91.0 | 50.0 | 89.9 |
| PP-high5 | 46.7 | 6.43 | 42.3 | 6.43 | 9.42 | 96.1 | 91.2 | 49.0 | 90.7 |

^1^ PR-low = prepartum low SCC; PR-high = prepartum high SCC; PP-low = postpartum low SCC; PP-high = postpartum high SCC.

**Table S3**. Gene ontology analysis of differentially expressed genes in the neutrophils of cows with low or high somatic cell counts at Week 1 prepartum.

| GOID | GO Term | *P*-Value | Genes Ratio (%) | Gene Count |
| --- | --- | --- | --- | --- |
| ***Biological Process-Up*** | | | | |
| GO:0022402 | cell cycle process | 2.2802E-24 | 9.97 | 96 |
| GO:0051276 | chromosome organization | 2.2630E-22 | 9.59 | 93 |
| GO:0000278 | mitotic cell cycle | 1.6128E-21 | 10.66 | 78 |
| GO:0006260 | DNA replication | 2.8208E-20 | 20.32 | 38 |
| GO:0098813 | nuclear chromosome segregation | 6.3688E-20 | 17.72 | 42 |
| GO:1903047 | mitotic cell cycle process | 2.8226E-19 | 11.43 | 64 |
| GO:0000819 | sister chromatid segregation | 4.8105E-19 | 20.71 | 35 |
| GO:0006259 | DNA metabolic process | 2.3949E-18 | 10.11 | 71 |
| GO:0000070 | mitotic sister chromatid segregation | 6.8794E-18 | 22.14 | 31 |
| GO:0000280 | nuclear division | 1.3572E-16 | 13.29 | 46 |
| GO:0140014 | mitotic nuclear division | 7.8999E-16 | 16.07 | 36 |
| GO:0022613 | ribonucleoprotein complex biogenesis | 2.0156E-15 | 12.64 | 45 |
| GO:0048285 | organelle fission | 2.2001E-15 | 12.14 | 47 |
| GO:0042254 | ribosome biogenesis | 2.7791E-15 | 15.04 | 37 |
| GO:0006261 | DNA-dependent DNA replication | 4.6883E-15 | 21.85 | 26 |
| GO:0034641 | cellular nitrogen compound metabolic process | 3.8569E-14 | 4.79 | 236 |
| GO:0006996 | organelle organization | 3.9386E-14 | 5.47 | 169 |
| GO:0006281 | DNA repair | 1.3382E-13 | 11.08 | 46 |
| GO:0006974 | cellular response to DNA damage stimulus | 2.7393E-11 | 8.40 | 56 |
| GO:0071897 | DNA biosynthetic process | 3.6136E-11 | 17.16 | 23 |
| GO:0034660 | ncRNA metabolic process | 3.6836E-11 | 10.44 | 40 |
| GO:0051726 | regulation of cell cycle | 6.5971E-11 | 7.58 | 65 |
| GO:0071103 | DNA conformation change | 8.5646E-11 | 13.59 | 28 |
| GO:0032392 | DNA geometric change | 9.1072E-11 | 23.29 | 17 |
| GO:0042273 | ribosomal large subunit biogenesis | 4.4109E-10 | 22.86 | 16 |
| GO:0044786 | cell cycle DNA replication | 8.4706E-10 | 32.43 | 12 |
| GO:0034470 | ncRNA processing | 9.6800E-10 | 10.75 | 33 |
| GO:0006139 | nucleobase-containing compound metabolic process | 1.1435E-09 | 4.62 | 198 |
| GO:0046483 | heterocycle metabolic process | 2.0399E-09 | 4.57 | 201 |
| GO:0032508 | DNA duplex unwinding | 2.1196E-09 | 22.39 | 15 |
| GO:0140013 | meiotic nuclear division | 2.1678E-09 | 14.67 | 22 |
| GO:0003678 | DNA helicase activity | 5.1841E-09 | 22.95 | 14 |
| GO:0006725 | cellular aromatic compound metabolic process | 6.1139E-09 | 4.51 | 201 |
| GO:0044249 | cellular biosynthetic process | 6.4160E-09 | 4.51 | 202 |
| GO:1903046 | meiotic cell cycle process | 6.5292E-09 | 13.29 | 23 |
| GO:0090304 | nucleic acid metabolic process | 7.0489E-09 | 4.64 | 182 |
| GO:1901576 | organic substance biosynthetic process | 7.5951E-09 | 4.48 | 204 |
| GO:0009059 | macromolecule biosynthetic process | 1.2165E-08 | 4.66 | 176 |
| GO:0006412 | translation | 1.6736E-08 | 8.13 | 43 |
| GO:1901360 | organic cyclic compound metabolic process | 2.0488E-08 | 4.44 | 204 |
| GO:0006364 | rRNA processing | 2.3019E-08 | 12.94 | 22 |
| GO:0000226 | microtubule cytoskeleton organization | 3.3920E-08 | 8.15 | 41 |
| GO:0007051 | spindle organization | 4.3008E-08 | 13.61 | 20 |
| GO:0034645 | cellular macromolecule biosynthetic process | 4.4470E-08 | 4.62 | 170 |
| GO:0051983 | regulation of chromosome segregation | 4.7158E-08 | 16.84 | 16 |
| GO:0043043 | peptide biosynthetic process | 5.4149E-08 | 7.90 | 43 |
| GO:0051383 | kinetochore organization | 5.5140E-08 | 42.11 | 8 |
| GO:0016072 | rRNA metabolic process | 5.9156E-08 | 12.29 | 22 |
| GO:0051310 | metaphase plate congression | 9.5847E-08 | 22.22 | 12 |
| GO:0051321 | meiotic cell cycle | 1.2311E-07 | 11.39 | 23 |
| GO:0045132 | meiotic chromosome segregation | 1.9957E-07 | 17.50 | 14 |
| GO:0051303 | establishment of chromosome localization | 2.2215E-07 | 18.84 | 13 |
| GO:0034061 | DNA polymerase activity | 2.2215E-07 | 18.84 | 13 |
| GO:0043604 | amide biosynthetic process | 2.4216E-07 | 7.17 | 46 |
| GO:0000723 | telomere maintenance | 2.6073E-07 | 14.95 | 16 |
| GO:0032200 | telomere organization | 2.6073E-07 | 14.95 | 16 |
| GO:0050000 | chromosome localization | 2.6501E-07 | 18.57 | 13 |
| GO:0006518 | peptide metabolic process | 2.8216E-07 | 7.14 | 47 |
| GO:0044260 | cellular macromolecule metabolic process | 3.5650E-07 | 4.02 | 264 |
| GO:0034622 | cellular protein-containing complex assembly | 3.7607E-07 | 6.56 | 54 |
| GO:0051304 | chromosome separation | 4.3466E-07 | 16.47 | 14 |
| GO:0033260 | nuclear DNA replication | 4.5414E-07 | 28.13 | 9 |
| GO:0006310 | DNA recombination | 5.4391E-07 | 10.17 | 24 |
| GO:0065004 | protein-DNA complex assembly | 5.4619E-07 | 12.18 | 19 |
| GO:0010564 | regulation of cell cycle process | 7.3464E-07 | 7.74 | 37 |
| GO:0044271 | cellular nitrogen compound biosynthetic process | 7.7691E-07 | 4.49 | 164 |
| GO:0009411 | response to UV | 8.0304E-07 | 13.79 | 16 |
| GO:0006275 | regulation of DNA replication | 8.3731E-07 | 16.88 | 13 |
| GO:0042255 | ribosome assembly | 8.4074E-07 | 20.37 | 11 |
| GO:0006399 | tRNA metabolic process | 8.9102E-07 | 11.80 | 19 |
| GO:0000027 | ribosomal large subunit assembly | 9.3618E-07 | 30.77 | 8 |
| GO:0045786 | negative regulation of cell cycle | 9.3673E-07 | 8.38 | 31 |
| GO:0044772 | mitotic cell cycle phase transition | 1.0696E-06 | 9.06 | 27 |
| GO:0043933 | protein-containing complex subunit organization | 1.6716E-06 | 5.57 | 74 |
| GO:0090307 | mitotic spindle assembly | 2.1432E-06 | 20.83 | 10 |
| GO:0043603 | cellular amide metabolic process | 2.9297E-06 | 6.20 | 53 |
| GO:0044770 | cell cycle phase transition | 2.9541E-06 | 8.41 | 28 |
| GO:0007346 | regulation of mitotic cell cycle | 3.0371E-06 | 7.59 | 34 |
| GO:1902850 | microtubule cytoskeleton organization involved in mitosis | 3.2372E-06 | 13.16 | 15 |
| GO:0022618 | ribonucleoprotein complex assembly | 3.4476E-06 | 11.25 | 18 |
| GO:0065003 | protein-containing complex assembly | 3.6734E-06 | 5.67 | 67 |
| GO:0010833 | telomere maintenance via telomere lengthening | 4.1766E-06 | 17.46 | 11 |
| GO:0007080 | mitotic metaphase plate congression | 4.3956E-06 | 21.95 | 9 |
| GO:0007052 | mitotic spindle organization | 4.5253E-06 | 14.61 | 13 |
| GO:0033044 | regulation of chromosome organization | 4.9083E-06 | 8.55 | 26 |
| GO:0051225 | spindle assembly | 5.1383E-06 | 14.44 | 13 |
| GO:0090329 | regulation of DNA-dependent DNA replication | 5.4383E-06 | 21.43 | 9 |
| GO:0071826 | ribonucleoprotein complex subunit organization | 5.8223E-06 | 10.84 | 18 |
| GO:0034508 | centromere complex assembly | 6.8449E-06 | 29.17 | 7 |
| GO:0045839 | negative regulation of mitotic nuclear division | 8.1709E-06 | 20.45 | 9 |
| GO:0022607 | cellular component assembly | 8.5815E-06 | 4.84 | 105 |
| GO:2000278 | regulation of DNA biosynthetic process | 9.0631E-06 | 14.81 | 12 |
| GO:0070192 | chromosome organization involved in meiotic cell cycle | 9.2877E-06 | 17.86 | 10 |
| GO:0007127 | meiosis I | 9.4415E-06 | 13.68 | 13 |
| GO:0009451 | RNA modification | 9.7923E-06 | 11.43 | 16 |
| GO:0071824 | protein-DNA complex subunit organization | 1.1357E-05 | 9.95 | 19 |
| GO:0000727 | double-strand break repair via break-induced replication | 1.2605E-05 | 45.45 | 5 |
| GO:0070925 | organelle assembly | 1.2863E-05 | 6.43 | 43 |
| GO:2000816 | negative regulation of mitotic sister chromatid separation | 1.3701E-05 | 22.22 | 8 |
| GO:0010032 | meiotic chromosome condensation | 1.4444E-05 | 66.67 | 4 |
| GO:1905819 | negative regulation of chromosome separation | 1.6999E-05 | 21.62 | 8 |
| GO:0045787 | positive regulation of cell cycle | 1.7477E-05 | 8.33 | 24 |
| GO:1901566 | organonitrogen compound biosynthetic process | 1.8543E-05 | 5.31 | 71 |
| GO:0061982 | meiosis I cell cycle process | 1.8563E-05 | 12.87 | 13 |
| GO:0033554 | cellular response to stress | 2.0896E-05 | 5.17 | 76 |
| GO:0033045 | regulation of sister chromatid segregation | 2.3555E-05 | 14.67 | 11 |
| GO:0033048 | negative regulation of mitotic sister chromatid segregation | 2.5615E-05 | 20.51 | 8 |
| GO:0003887 | DNA-directed DNA polymerase activity | 2.6886E-05 | 24.14 | 7 |
| GO:0000470 | maturation of LSU-rRNA | 2.6886E-05 | 24.14 | 7 |
| GO:0003887 | DNA-directed DNA polymerase activity | 2.6886E-05 | 24.14 | 7 |
| GO:2000573 | positive regulation of DNA biosynthetic process | 2.8774E-05 | 17.65 | 9 |
| GO:0000725 | recombinational repair | 3.0433E-05 | 11.57 | 14 |
| GO:0010948 | negative regulation of cell cycle process | 3.0504E-05 | 10.00 | 17 |
| GO:0033046 | negative regulation of sister chromatid segregation | 3.1136E-05 | 20.00 | 8 |
| GO:0036388 | pre-replicative complex assembly | 3.2854E-05 | 57.14 | 4 |
| GO:0006267 | pre-replicative complex assembly involved in nuclear cell cycle DNA replication | 3.2854E-05 | 57.14 | 4 |
| GO:1902299 | pre-replicative complex assembly involved in cell cycle DNA replication | 3.2854E-05 | 57.14 | 4 |
| GO:0051784 | negative regulation of nuclear division | 3.3828E-05 | 17.31 | 9 |
| GO:0008608 | attachment of spindle microtubules to kinetochore | 3.4114E-05 | 23.33 | 7 |
| GO:0006278 | RNA-dependent DNA biosynthetic process | 3.6144E-05 | 15.38 | 10 |
| GO:0051985 | negative regulation of chromosome segregation | 3.7619E-05 | 19.51 | 8 |
| GO:0090068 | positive regulation of cell cycle process | 4.2992E-05 | 9.05 | 19 |
| GO:0034502 | protein localization to chromosome | 4.3670E-05 | 13.75 | 11 |
| GO:0010965 | regulation of mitotic sister chromatid separation | 4.6216E-05 | 16.67 | 9 |
| GO:0006301 | postreplication repair | 4.8668E-05 | 27.27 | 6 |
| GO:0051382 | kinetochore assembly | 5.0451E-05 | 35.71 | 5 |
| GO:0007076 | mitotic chromosome condensation | 5.0451E-05 | 35.71 | 5 |
| GO:0097472 | cyclin-dependent protein kinase activity | 5.1992E-05 | 12.50 | 12 |
| GO:0007004 | telomere maintenance via telomerase | 5.3722E-05 | 16.36 | 9 |
| GO:2001251 | negative regulation of chromosome organization | 5.6252E-05 | 11.61 | 13 |
| GO:0051306 | mitotic sister chromatid separation | 6.2231E-05 | 16.07 | 9 |
| GO:0006335 | DNA replication-dependent nucleosome assembly | 6.4054E-05 | 50.00 | 4 |
| GO:0034723 | DNA replication-dependent nucleosome organization | 6.4054E-05 | 50.00 | 4 |
| GO:0009303 | rRNA transcription | 6.4086E-05 | 26.09 | 6 |
| GO:0006396 | RNA processing | 6.4761E-05 | 6.03 | 43 |
| GO:0006323 | DNA packaging | 6.7722E-05 | 10.77 | 14 |
| GO:0051052 | regulation of DNA metabolic process | 6.8025E-05 | 8.03 | 22 |
| GO:0000463 | maturation of LSU-rRNA from tricistronic rRNA transcript (SSU-rRNA, 5.8S rRNA, LSU-rRNA) | 7.3703E-05 | 33.33 | 5 |
| GO:0045841 | negative regulation of mitotic metaphase/anaphase transition | 8.0741E-05 | 20.59 | 7 |
| GO:1905818 | regulation of chromosome separation | 9.4856E-05 | 15.25 | 9 |
| GO:1902100 | negative regulation of metaphase/anaphase transition of cell cycle | 9.8186E-05 | 20.00 | 7 |
| GO:0009314 | response to radiation | 1.1035E-04 | 7.59 | 24 |
| GO:0000724 | double-strand break repair via homologous recombination | 1.1514E-04 | 10.83 | 13 |
| GO:0032875 | regulation of DNA endoreduplication | 1.2511E-04 | 75.00 | 3 |
| ***Cellular Component-Up*** | | | | |
| GO:0043232 | intracellular non-membrane-bounded organelle | 2.8434E-28 | 6.29 | 218 |
| GO:0043228 | non-membrane-bounded organelle | 2.9976E-28 | 6.29 | 218 |
| GO:0070013 | intracellular organelle lumen | 3.5562E-24 | 5.88 | 219 |
| GO:0043233 | organelle lumen | 3.5562E-24 | 5.88 | 219 |
| GO:0005694 | chromosome | 9.4833E-22 | 9.81 | 87 |
| GO:0005634 | nucleus | 2.5958E-21 | 5.00 | 278 |
| GO:0031981 | nuclear lumen | 7.8391E-21 | 5.86 | 197 |
| GO:0005730 | nucleolus | 4.2006E-18 | 9.94 | 71 |
| GO:0043229 | intracellular organelle | 5.9035E-18 | 4.06 | 398 |
| GO:0043231 | intracellular membrane-bounded organelle | 2.6851E-16 | 4.23 | 350 |
| GO:0000793 | condensed chromosome | 5.4652E-16 | 19.02 | 31 |
| GO:0005654 | nucleoplasm | 1.7411E-15 | 5.68 | 164 |
| GO:0098687 | chromosomal region | 4.7717E-13 | 13.36 | 35 |
| GO:0043227 | membrane-bounded organelle | 1.0797E-12 | 4.01 | 358 |
| GO:0000775 | chromosome, centromeric region | 3.9287E-12 | 15.88 | 27 |
| GO:0000776 | kinetochore | 1.8241E-11 | 18.49 | 22 |
| GO:0000779 | condensed chromosome, centromeric region | 5.1727E-11 | 22.22 | 18 |
| GO:0000228 | nuclear chromosome | 1.4318E-10 | 9.15 | 45 |
| GO:0000777 | condensed chromosome kinetochore | 7.3473E-09 | 22.22 | 14 |
| GO:0015630 | microtubule cytoskeleton | 8.0983E-09 | 6.59 | 67 |
| GO:0000796 | condensin complex | 2.5465E-08 | 75.00 | 6 |
| GO:0005739 | mitochondrion | 1.0738E-07 | 5.90 | 76 |
| GO:0030684 | preribosome | 1.6943E-07 | 19.12 | 13 |
| GO:0005819 | spindle | 2.3221E-07 | 9.71 | 27 |
| GO:0000794 | condensed nuclear chromosome | 2.4938E-07 | 17.07 | 14 |
| GO:0005813 | centrosome | 6.9671E-07 | 7.55 | 38 |
| GO:0005657 | replication fork | 7.5298E-07 | 18.46 | 12 |
| GO:0005732 | small nucleolar ribonucleoprotein complex | 1.0842E-06 | 36.84 | 7 |
| GO:0005815 | microtubule organizing center | 2.5902E-06 | 6.74 | 43 |
| GO:0030687 | preribosome, large subunit precursor | 4.7262E-06 | 30.43 | 7 |
| GO:0005759 | mitochondrial matrix | 1.2189E-05 | 8.45 | 24 |
| GO:0000940 | condensed chromosome outer kinetochore | 2.0233E-05 | 41.67 | 5 |
| GO:0005829 | cytosol | 2.8373E-05 | 4.46 | 125 |
| GO:0001650 | fibrillar center | 5.1859E-05 | 11.61 | 13 |
| GO:0031298 | replication fork protection complex | 6.2043E-05 | 50.00 | 4 |
| GO:0005840 | ribosome | 6.4346E-05 | 8.72 | 19 |
| GO:0031429 | box H/ACA snoRNP complex | 1.2209E-04 | 75.00 | 3 |
| GO:0090661 | box H/ACA telomerase RNP complex | 1.2209E-04 | 75.00 | 3 |
| GO:0072588 | box H/ACA RNP complex | 1.2209E-04 | 75.00 | 3 |
| GO:0031262 | Ndc80 complex | 1.2209E-04 | 75.00 | 3 |
| GO:0031617 | NMS complex | 1.2209E-04 | 75.00 | 3 |
| GO:0005874 | microtubule | 1.2595E-04 | 7.64 | 22 |
| GO:0043596 | nuclear replication fork | 1.9144E-04 | 17.95 | 7 |
| GO:0031428 | box C/D snoRNP complex | 2.9806E-04 | 60.00 | 3 |
| GO:0005856 | cytoskeleton | 3.1410E-04 | 4.67 | 80 |
| GO:0072686 | mitotic spindle | 4.7607E-04 | 10.48 | 11 |
| GO:0045171 | intercellular bridge | 5.0152E-04 | 13.56 | 8 |
| ***Molecular Function-Up*** | | | | |
| GO:0003723 | RNA binding | 8.4671E-12 | 7.47 | 71 |
| GO:0003676 | nucleic acid binding | 9.9851E-12 | 5.22 | 157 |
| GO:0000166 | nucleotide binding | 9.5411E-10 | 5.66 | 108 |
| GO:1901265 | nucleoside phosphate binding | 9.5411E-10 | 5.66 | 108 |
| GO:0003678 | DNA helicase activity | 4.8007E-09 | 22.95 | 14 |
| GO:0030554 | adenyl nucleotide binding | 9.4122E-09 | 6.05 | 81 |
| GO:0032559 | adenyl ribonucleotide binding | 1.5256E-08 | 6.01 | 80 |
| GO:0035639 | purine ribonucleoside triphosphate binding | 1.7655E-08 | 5.67 | 92 |
| GO:0017076 | purine nucleotide binding | 2.1007E-08 | 5.60 | 95 |
| GO:0005524 | ATP binding | 2.2431E-08 | 6.05 | 78 |
| GO:0032553 | ribonucleotide binding | 2.2530E-08 | 5.58 | 95 |
| GO:0042623 | ATPase activity, coupled | 2.6449E-08 | 10.80 | 27 |
| GO:0032555 | purine ribonucleotide binding | 3.1399E-08 | 5.57 | 94 |
| GO:0016887 | ATPase activity | 3.8604E-08 | 8.56 | 37 |
| GO:0016779 | nucleotidyltransferase activity | 1.0625E-07 | 12.82 | 20 |
| GO:0034061 | DNA polymerase activity | 2.0750E-07 | 18.84 | 13 |
| GO:0017116 | single-stranded DNA helicase activity | 2.7172E-07 | 43.75 | 7 |
| GO:0140101 | catalytic activity, acting on a tRNA | 4.0246E-07 | 14.41 | 16 |
| GO:0019899 | enzyme binding | 2.7319E-06 | 5.13 | 90 |
| GO:0043168 | anion binding | 3.4777E-06 | 4.75 | 117 |
| GO:0016818 | hydrolase activity, acting on acid anhydrides, in phosphorus-containing anhydrides | 4.9307E-06 | 5.60 | 66 |
| GO:0016817 | hydrolase activity, acting on acid anhydrides | 5.0585E-06 | 5.59 | 66 |
| GO:0001094 | TFIID-class transcription factor complex binding | 6.8710E-06 | 50.00 | 5 |
| GO:0016462 | pyrophosphatase activity | 7.6943E-06 | 5.54 | 65 |
| GO:0001091 | RNA polymerase II general transcription initiation factor binding | 8.9533E-06 | 35.29 | 6 |
| GO:0003887 | DNA-directed DNA polymerase activity | 2.5929E-05 | 24.14 | 7 |
| GO:0003697 | single-stranded DNA binding | 2.8635E-05 | 13.19 | 12 |
| GO:0097472 | cyclin-dependent protein kinase activity | 4.9152E-05 | 12.50 | 12 |
| GO:0043138 | 3'-5' DNA helicase activity | 1.0171E-04 | 31.25 | 5 |
| GO:0070182 | DNA polymerase binding | 1.0171E-04 | 31.25 | 5 |
| GO:0017111 | nucleoside-triphosphatase activity | 1.1601E-04 | 5.26 | 59 |
| GO:0034513 | box H/ACA snoRNA binding | 1.2310E-04 | 75.00 | 3 |
| GO:0001042 | RNA polymerase I core binding | 1.2310E-04 | 75.00 | 3 |
| GO:0030515 | snoRNA binding | 1.3071E-04 | 23.08 | 6 |
| GO:1904029 | regulation of cyclin-dependent protein kinase activity | 1.3458E-04 | 13.16 | 10 |
| GO:0003899 | DNA-directed 5'-3' RNA polymerase activity | 1.3740E-04 | 18.92 | 7 |
| GO:0016741 | transferase activity, transferring one-carbon groups | 1.3901E-04 | 8.81 | 17 |
| GO:0004693 | cyclin-dependent protein serine/threonine kinase activity | 1.5122E-04 | 11.96 | 11 |
| GO:0034062 | 5'-3' RNA polymerase activity | 1.6389E-04 | 18.42 | 7 |
| GO:0097747 | RNA polymerase activity | 1.6389E-04 | 18.42 | 7 |
| GO:0003677 | DNA binding | 2.7909E-04 | 4.66 | 83 |
| ***Biological Process-Down*** | | | | |
| GO:0009615 | response to virus | 3.6650E-09 | 10.82 | 25 |
| GO:0007166 | cell surface receptor signaling pathway | 8.0508E-09 | 4.74 | 101 |
| GO:0070887 | cellular response to chemical stimulus | 1.4309E-08 | 4.61 | 105 |
| GO:0035556 | intracellular signal transduction | 1.9242E-08 | 4.72 | 97 |
| GO:0071310 | cellular response to organic substance | 1.0909E-07 | 4.76 | 86 |
| GO:0034097 | response to cytokine | 1.5309E-07 | 6.38 | 43 |
| GO:0045064 | T-helper 2 cell differentiation | 3.0009E-07 | 50.00 | 6 |
| GO:0046631 | alpha-beta T cell activation | 3.2817E-07 | 13.39 | 15 |
| GO:0007159 | leukocyte cell-cell adhesion | 3.4963E-07 | 8.86 | 24 |
| GO:0045936 | negative regulation of phosphate metabolic process | 3.5652E-07 | 7.29 | 32 |
| GO:0010563 | negative regulation of phosphorus metabolic process | 3.5652E-07 | 7.29 | 32 |
| GO:0051707 | response to other organism | 3.5730E-07 | 5.70 | 51 |
| GO:0043207 | response to external biotic stimulus | 3.7788E-07 | 5.69 | 51 |
| GO:0010033 | response to organic substance | 5.1175E-07 | 4.46 | 95 |
| GO:0004896 | cytokine receptor activity | 5.5784E-07 | 14.94 | 13 |
| GO:0048513 | animal organ development | 6.6374E-07 | 4.23 | 109 |
| GO:0042326 | negative regulation of phosphorylation | 8.5902E-07 | 7.78 | 27 |
| GO:0023051 | regulation of signaling | 9.2175E-07 | 4.21 | 108 |
| GO:0030154 | cell differentiation | 1.1696E-06 | 4.05 | 122 |
| GO:0031400 | negative regulation of protein modification process | 1.2600E-06 | 6.88 | 32 |
| GO:0048583 | regulation of response to stimulus | 1.4166E-06 | 4.05 | 120 |
| GO:0046634 | regulation of alpha-beta T cell activation | 1.5991E-06 | 16.42 | 11 |
| GO:0045628 | regulation of T-helper 2 cell differentiation | 1.6105E-06 | 55.56 | 5 |
| GO:0006952 | defense response | 1.7656E-06 | 5.30 | 54 |
| GO:0030097 | hemopoiesis | 1.9266E-06 | 6.02 | 40 |
| GO:0098542 | defense response to other organism | 2.0529E-06 | 6.01 | 40 |
| GO:0016477 | cell migration | 2.2474E-06 | 5.11 | 58 |
| GO:0010646 | regulation of cell communication | 2.3558E-06 | 4.16 | 106 |
| GO:0071345 | cellular response to cytokine stimulus | 2.4702E-06 | 6.17 | 38 |
| GO:0009966 | regulation of signal transduction | 2.6379E-06 | 4.29 | 96 |
| GO:0051607 | defense response to virus | 2.7482E-06 | 9.73 | 18 |
| GO:0019221 | cytokine-mediated signaling pathway | 3.2424E-06 | 7.43 | 26 |
| GO:0048534 | hematopoietic or lymphoid organ development | 3.4711E-06 | 5.84 | 41 |
| GO:0032101 | regulation of response to external stimulus | 3.4711E-06 | 5.84 | 41 |
| GO:0048534 | hematopoietic or lymphoid organ development | 3.4711E-06 | 5.84 | 41 |
| GO:0098609 | cell-cell adhesion | 3.9398E-06 | 6.12 | 37 |
| GO:0048584 | positive regulation of response to stimulus | 4.4403E-06 | 4.54 | 76 |
| GO:1903706 | regulation of hemopoiesis | 4.8540E-06 | 7.85 | 23 |
| GO:1902531 | regulation of intracellular signal transduction | 5.0034E-06 | 4.92 | 60 |
| GO:0001933 | negative regulation of protein phosphorylation | 5.0483E-06 | 7.62 | 24 |
| GO:0002520 | immune system development | 6.7585E-06 | 5.64 | 42 |
| GO:0009967 | positive regulation of signal transduction | 7.2927E-06 | 4.88 | 59 |
| GO:0048585 | negative regulation of response to stimulus | 8.1443E-06 | 4.88 | 60 |
| GO:0045580 | regulation of T cell differentiation | 8.2449E-06 | 11.82 | 13 |
| GO:0010604 | positive regulation of macromolecule metabolic process | 8.3194E-06 | 4.03 | 110 |
| GO:0002292 | T cell differentiation involved in immune response | 1.0771E-05 | 16.98 | 9 |
| GO:2000514 | regulation of CD4-positive, alpha-beta T cell activation | 1.1504E-05 | 19.51 | 8 |
| GO:0023056 | positive regulation of signaling | 1.2140E-05 | 4.71 | 63 |
| GO:0035455 | response to interferon-alpha | 1.5040E-05 | 38.46 | 5 |
| GO:0042325 | regulation of phosphorylation | 1.5413E-05 | 4.78 | 60 |
| GO:0035710 | CD4-positive, alpha-beta T cell activation | 1.9520E-05 | 14.08 | 10 |
| GO:1902105 | regulation of leukocyte differentiation | 1.9546E-05 | 8.45 | 18 |
| GO:0046632 | alpha-beta T cell differentiation | 2.1488E-05 | 12.64 | 11 |
| GO:0030099 | myeloid cell differentiation | 2.2655E-05 | 7.33 | 22 |
| GO:0048870 | cell motility | 2.2711E-05 | 4.72 | 59 |
| GO:1902107 | positive regulation of leukocyte differentiation | 2.3340E-05 | 10.74 | 13 |
| GO:0019220 | regulation of phosphate metabolic process | 2.7279E-05 | 4.57 | 64 |
| GO:0051174 | regulation of phosphorus metabolic process | 2.7521E-05 | 4.57 | 64 |
| GO:0046637 | regulation of alpha-beta T cell differentiation | 3.2863E-05 | 17.02 | 8 |
| GO:0002294 | CD4-positive, alpha-beta T cell differentiation involved in immune response | 3.2863E-05 | 17.02 | 8 |
| GO:0002293 | alpha-beta T cell differentiation involved in immune response | 3.8517E-05 | 16.67 | 8 |
| GO:0002287 | alpha-beta T cell activation involved in immune response | 3.8517E-05 | 16.67 | 8 |
| GO:0010647 | positive regulation of cell communication | 3.8914E-05 | 4.59 | 61 |
| GO:0038061 | NIK/NF-kappaB signaling | 4.0472E-05 | 11.83 | 11 |
| GO:0031399 | regulation of protein modification process | 4.2340E-05 | 4.47 | 66 |
| GO:0045622 | regulation of T-helper cell differentiation | 4.2682E-05 | 24.00 | 6 |
| GO:0030217 | T cell differentiation | 4.4827E-05 | 8.25 | 17 |
| GO:0046633 | alpha-beta T cell proliferation | 5.4232E-05 | 23.08 | 6 |
| GO:0002064 | epithelial cell development | 6.2503E-05 | 8.38 | 16 |
| GO:0031324 | negative regulation of cellular metabolic process | 6.8131E-05 | 4.11 | 84 |
| GO:0045619 | regulation of lymphocyte differentiation | 7.4030E-05 | 9.63 | 13 |
| GO:0045630 | positive regulation of T-helper 2 cell differentiation | 7.6505E-05 | 75.00 | 3 |
| GO:1901222 | regulation of NIK/NF-kappaB signaling | 7.7126E-05 | 12.05 | 10 |
| GO:0002682 | regulation of immune system process | 7.8573E-05 | 4.81 | 49 |
| ***Molecular Function-Down*** | | | | |
| GO:0140375 | immune receptor activity | 5.9064E-10 | 17.17 | 17 |
| GO:0004896 | cytokine receptor activity | 3.4757E-07 | 14.94 | 13 |
| GO:0019955 | cytokine binding | 2.0739E-05 | 11.21 | 12 |
| GO:1901981 | phosphatidylinositol phosphate binding | 7.6193E-05 | 9.22 | 13 |


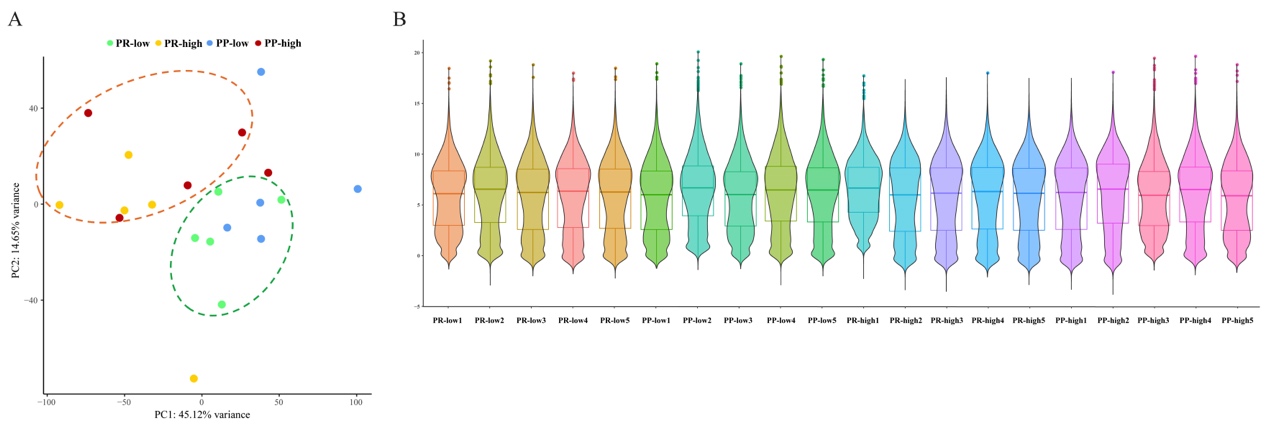
**Figure S1**. Transcriptomic profiles of neutrophils in dairy cows with high or low somatic cell counts at Week 1 pre- and postpartum. (A) Principal component analysis; (B) The violin plot of gene expression dispersion among samples. PR-low = prepartum low SCC; PP-low = postpartum low SCC; PR-high = prepartum high SCC; PP-high = postpartum high SCC.
